# Biocompatibility and cytotoxicity in vitro of surface-functionalized drug-loaded spinel ferrite nanoparticles

**DOI:** 10.3762/bjnano.12.99

**Published:** 2021-12-02

**Authors:** Sadaf Mushtaq, Khuram Shahzad, Tariq Saeed, Anwar Ul-Hamid, Bilal Haider Abbasi, Nafees Ahmad, Waqas Khalid, Muhammad Atif, Zulqurnain Ali, Rashda Abbasi

**Affiliations:** 1 Institute of Biomedical and Genetic Engineering, G-9/1, Islamabad, Pakistan; 2 Department of Biotechnology, Quaid-i-Azam University, Islamabad, Pakistan; 3 Department of Physics, Functional Materials Lab, Air University, Sector E-9, Islamabad, Pakistan; 4 Core Research Facilities, King Fahd University of Petroleum & Minerals, Dhahran 31261, Saudi Arabia

**Keywords:** anticancer drugs, doxorubicin, drug carriers, in vitro studies, magnetic spinel ferrite nanoparticles, methotrexate, poly(isobutylene-*alt*-maleic anhydride)

## Abstract

In this study, poly(isobutylene-*alt*-maleic anhydride) (PMA)-coated spinel ferrite (MFe_2_O_4_, where M = Fe, Co, Ni, or Zn) nanoparticles (NPs) were developed as carriers of the anticancer drugs doxorubicin (DOX) and methotrexate (MTX). Physical characterizations confirmed the formation of pure cubic structures (14–22 nm) with magnetic properties. Drug-loaded NPs exhibited tumor specificity with significantly higher (*p* < 0.005) drug release in an acidic environment (pH 5.5). The nanoparticles were highly colloidal (zeta potential = −35 to −26 mV) in deionized water, phosphate buffer saline (PBS), and sodium borate buffer (SBB). They showed elevated and dose-dependent cytotoxicity in vitro compared to free drug controls. The IC_50_ values ranged from 0.81 to 3.97 μg/mL for HepG2 and HT144 cells, whereas IC_50_ values for normal lymphocytes were 10 to 35 times higher (18.35–43.04 µg/mL). Cobalt ferrite (CFO) and zinc ferrite (ZFO) NPs were highly genotoxic (*p* < 0.05) in cancer cell lines. The nanoparticles caused cytotoxicity via oxidative stress, causing DNA damage and activation of p53-mediated cell cycle arrest (significantly elevated expression, *p* < 0.005, majorly G1 and G2/M arrest) and apoptosis. Cytotoxicity testing in 3D spheroids showed significant (*p* < 0.05) reduction in spheroid diameter and up to 74 ± 8.9% of cell death after two weeks. In addition, they also inhibited multidrug resistance (MDR) pump activity in both cell lines suggesting effectivity in MDR cancers. Among the tested MFe_2_O_4_ NPs, CFO nanocarriers were the most favorable for targeted cancer therapy due to excellent magnetic, colloidal, cytotoxic, and biocompatible aspects. However, detailed mechanistic, in vivo cytotoxicity, and magnetic-field-assisted studies are required to fully exploit these nanocarriers in therapeutic applications.

## Introduction

Cancer is the second leading cause of death and, as such, it is a global health concern [1]. It is caused by uncontrolled cell proliferation, reduced cell death rate, or both [2]. Conventional treatment strategies for cancer, including surgery, radiotherapy, and chemotherapy, lack the ability to selectively target neoplastic tissue, which results in systemic toxicity [3]. For these reasons, the focus of the field was transferred to nanomedicine which enables targeted therapy and reduces side effects of conventional therapeutic agents [4]. Functionalized nanoparticles have the potential to improve the therapeutic performance of drugs by regulating pharmacokinetics and pharmacodynamics [5]. Moreover, water compatibility of nanocarriers provides better chemical stability and bioavailability of the encapsulated drug which allows controlled release. Additionally, the attached drug is protected from degradation, which allows an increased circulation time [6]. The targeting of specific tumor tissue is therefore achieved by an increased biodistribution process known as enhanced permeability and retention (EPR) effect [7].

Magnetic nanoparticles (MNPs) have gained significant attention as effective drug delivery systems due to their distinct physiochemical attributes, high surface-to-volume ratio, and the possibility of surface functionalization [8]. Furthermore, magnetic-field-assisted control of the behavior of MNPs makes them suitable candidates for targeted drug delivery, hyperthermia, biosensors, magnetic resonance imaging (MRI), and magnetic separation [9–10]. Magnetite (Fe_3_O_4_) nanoparticles (NPs), belonging to the spinel ferrite class, are the most extensively studied MNPs for clinical applications and many of them have been approved by the Food and Drug Administration (FDA) agency. Their intended applications include hyperthermia, disease diagnosis, MRI contrasting agents, and improvement of iron deficiencies [11–12]. Aside from their useful applications, magnetite NPs have some serious limitations, such as chemical reactivity, rapid oxidation, particle agglomeration, and high surface energy which may affect their biocompatibility and performance [11]. Moreover, they have low magnetization at a smaller size and the presence of iron has been associated with adverse interactions with hemoglobin [13].

Magnetic spinel ferrites nanoparticles (MSFNPs) with a general formula of MFe_2_O_4_ (where M = divalent cation of Co, Ni, Zn, Mn, or Mg) are soft magnetic materials with a face-centered cubic structure [14]. Among those, cobalt ferrite NPs have a large magnetocrystalline anisotropy, high saturation magnetization, and coercivity even at room temperature as compared to others [15]. The substitution of metal cations M^+^ for cobalt, nickel, and zinc contributes to diverse magnetic properties, morphology, and size of iron oxide NPs [13,16] along with varied tissue penetration and hemocompatibility which can be useful for biomedical applications [12,17].

Furthermore, in order to be exploited in biomedical applications, NPs need to fulfill certain criteria which include water solubility, excellent colloidal stability, biocompatibility, and high saturation magnetization which enables controlled and nontoxic biological interactions [18]. The hydrophilicity of the nanocarriers is important, as native hydrophobic surfaces of NPs are rapidly opsonized by hydrophobic serum proteins [19]. For this, surface functionalization has a major role [18]. It alters the surface chemistry of NPs, thereby affecting their physiochemical and biological properties [11,20].

In the present work, we synthesized a variety of MFe_2_O_4_ (M = Co, Ni, and Zn) NPs using the sonochemical technique. Particle agglomeration was prevented by using oleic acid as the surfactant [21]. Phase change of hydrophobic NPs was achieved by the functionalization with an amphiphilic brush copolymer, poly(isobutylene-*alt*-maleic anhydride) (PMA) implanted with dodecylamine, which provides biocompatibility, colloidal stability, and hydrophilicity [22]. It is composed of hydrophobic side chains and the backbone of hydrophilic groups. The hydrophobic side chains interact with the hydrophobic surfactant (oleic acid) present on the surface of NPs, thereby exposing the hydrophilic end to interact with the aqueous environment and contributing towards a colloidal nanosuspension [23]. The surfaces of the NPs were further functionalized with anticancer drugs, such as doxorubicin (DOX) and methotrexate (MTX) via 1-ethyl-3-(3-dimethylaminopropyl)carbodiimide (EDC) chemistry. The samples were stored at room temperature for further experiments. Our aim was to compare the biocompatibility, colloidal stability, and in vitro cytotoxicity of these nanocarriers for potential anticancer drug delivery systems.

## Results and Discussion

### Physical characterizations

**Figure 1:**
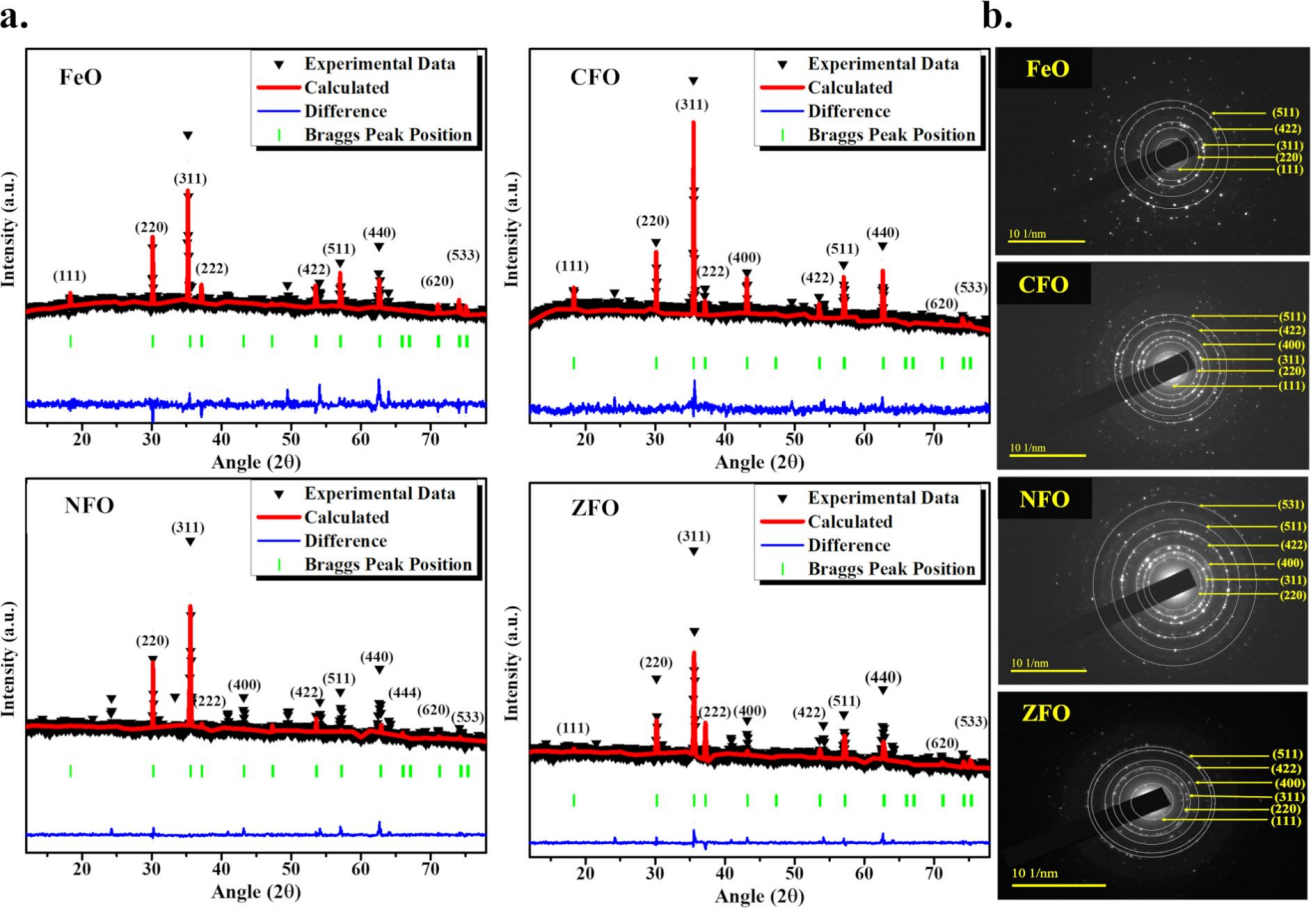
**(a)** Rietveld-refined XRD pattern of MFe_2_O_4_ (M = Fe, Co, Ni, Zn) NPs with SAED images showing different hkl planes. The triangles represent experimental points and the solid red line represents Rietveld-refined data. The bottom line (blue) shows the difference between the experimental and refined data. (b) The marked 2θ positions are the allowed Bragg peaks.

The X-ray diffraction (XRD) data of all samples was analyzed using Rietveld refinement techniques in the Fullprof Suit program. The data was refined according to their space groups. The Rietveld-refined XRD pattern of the MFe_2_O_4_ nanoparticles (Figure 1a), where triangles indicate experimental data, is shown by the red solid lines which represent the calculated intensities. The difference between the two intensities was indicated by the blue line at the bottom of the graphs and the positions of the Bragg peaks are marked with vertical green lines according to their space groups. All the observed peaks are allowed Bragg’s 2θ positions. The background was refined by using the pseudo-Voigt function and taking atomic fractional positions as fixed parameters during refinement. However, some factors such as lattice constant, isothermal parameters, scale, and shape factors were considered as free parameters. All the samples show low values of goodness of fit (χ^2^). Several physical parameters (e.g., lattice constant, average crystalline size, density) were calculated as given in Table 1. The prominent peaks originating from different planes (111, 220, 311, 222, 400, 422, 440, 533, 620), were found in good agreement with standard JCPDS cards (019-0629, 22-1086, 10-0325, and 82-1049 for M = Fe, Co, Ni, and Zn, respectively). A slight peak shift at the (311) plane was observed for cobalt ferrite (CFO), nickel ferrite (NFO), and zinc ferrite (ZFO) as compared to FeO due to the ionic radii difference of divalent M^+2^ cations. The peak shift also indicates the incorporation of M^+2^ cations into the lattice. Further confirmation of the crystalline nature of the composites was obtained by analyzing the selected area electron diffraction (SAED) patterns. The SAED images explain the position of the crystalline system upon diffraction. The results further provide the concentric rings that explains the different hkl planes, as shown in Figure 1b. Furthermore, the formation of the cubic phase of the samples is consistent with the XRD results.

**Table 1:** Different physical parameters calculated from XRD analysis.

Nanostructures	Crystallite size (nm)	Lattice constant (Å)	Goodness of fit (χ^2^)

FeO	27	8.43	2.03
CFO	23	8.39	2.11
NFO	33	8.35	2.53
ZFO	24	8.46	2.37

**Figure 2:**
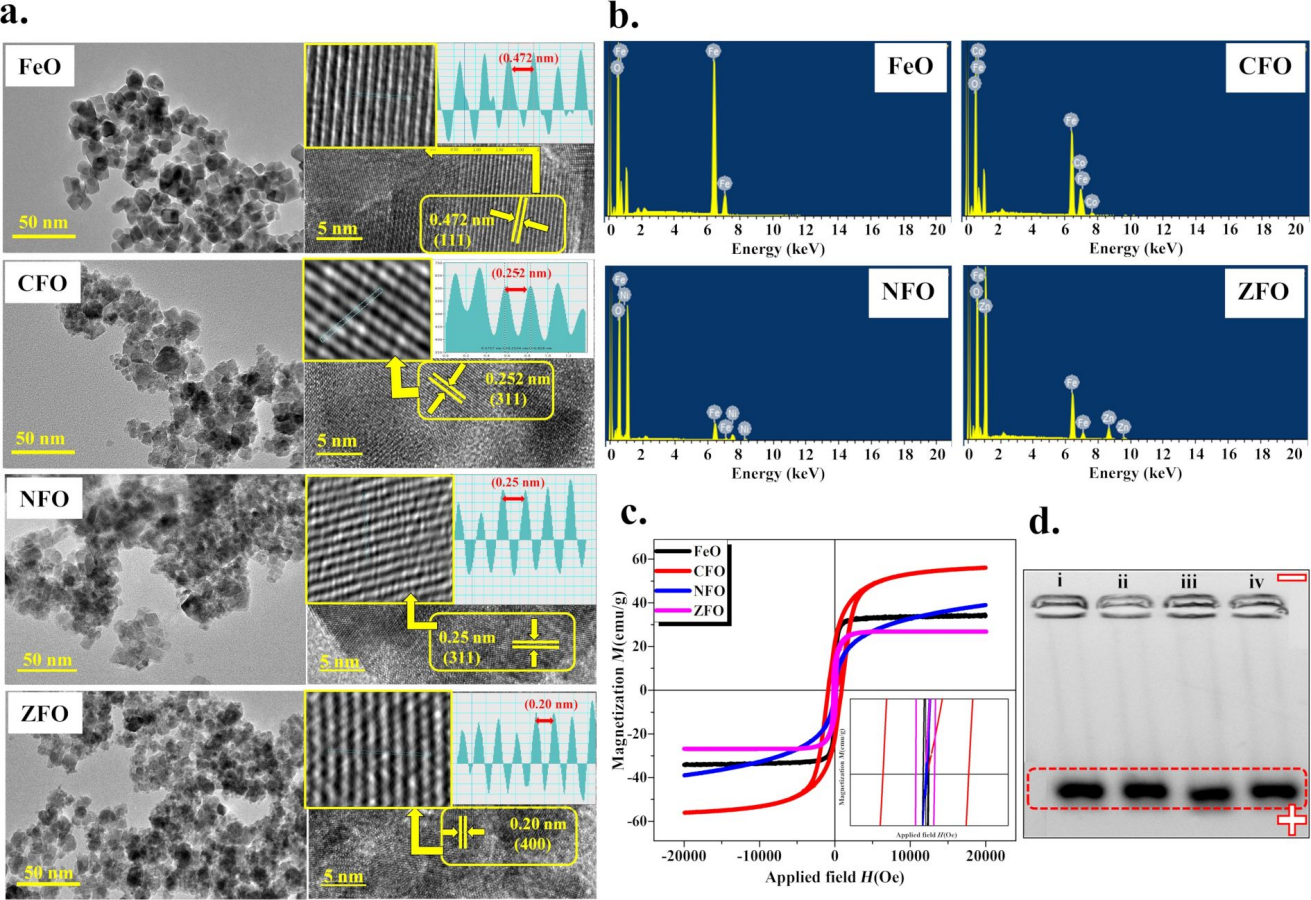
(a) HR-TEM micrographs of MFe_2_O_4_ (M = Fe, Co, Ni, Zn) NPs showing the respective planes. (b) EDS analysis showing major elemental composition in synthesized NPs. (c) Magnetization as a function of the applied field on NPs at room temperature at an applied field of 2.0 T. (d) Agarose gel electrophoresis image of (i) FeO-PMA, (ii) NFO-PMA, (iii) CFO-PMA, and (iv) ZFO-PMA NPs. The black bands on the gel indicate a uniform size distribution and a negative surface charge on colloidal NPs.

The formation of spherical NPs was confirmed by transmission electron microscopy (TEM) (Figure 2a). The nanospheres are uniformally distributed throughout the surface of the samples. High-resolution transmission electron microscopy (HR-TEM) images show a crystalline structure with edges of single grains of nanoparticles. The interplanar distance was meaured for each sample with marked lattice fringes of the respective planes. The average particle size was found to be 16–21 nm for FeO, 14–18 nm for CFO, and 12–16 nm for NFO with *d* spacing values of 0.47, 0.25, 0.24, and 0.20 nm for FeO, CFO, NFO and ZFO, respectively, corresponding to (111), (311), (311), and (400), respectively. These planes are well-matched with the interplanar distance of the diffraction pattern standards obtained from their standard JCPDS database.

Weight and atomic % of M = Fe ions in all samples, as studied by energy dispersive spectroscopy (EDS), is given in Table 2. No extra impurity peaks were present in the spectrum (Figure 2b) due to the use of coprecipitation synthesis methods in which the samples were washed several times to remove any impurity.

**Table 2:** EDS analysis showing elemental composition for MFe_2_O_4_ (M = Fe, Co, Ni, Zn) NPs.

	FeO		CFO			NFO			ZFO		
	
	O	Fe	O	Fe	Co	O	Fe	Ni	O	Fe	Zn

atomic %	63.66	36.34	63.30	24.28	12.43	63.26	24.20	12.55	69.38	20.41	10.21
weight %	33.42	66.58	36.76	46.29	16.96	37.50	48.33	14.17	40.22	42.86	16.91
total	100		100			100			100		

The physical property measurement system (PPMS) was used to evaluate magnetic properties of MFe_2_O_4_ NPs. Hysteresis loops were measured at room temperature upon an applied field of 2.0 T. The samples clearly showed ferromagnetic behavior with different saturation magnetization *M*_s_ (emu/g) and coercivity *H*_c_ (Oe) values, as shown in Table 3 [24]. From Figure 2c, all samples went through saturation at an applied field of 2.0 T, except nickel ferrite. This is may be due to the presence of a strong magnetic anisotropy, which required a higher applied field to induce saturation [25]. Cobalt ferrite has the maximum coercivity (883 Oe) and saturation magnetization values (56 emu/g) in comparison to other ferrites due to a high anisotropy. Also, during cationic distribution, Co^+2^ cations incorporate into Fe–O, whereas the cationic distribution of other divalent metals (e.g., Ni^+2^ or Zn^+2^) leads to a decrease in magnetic anisotropy [17,26]. Moreover, zinc ferrite has a slightly increased coercivity than nickel ferrite and iron oxide due to the formation of a noncollinear ferrimagnetic structure [27]. From Table 3, cobalt ferrite has the best magnetic properties in terms of saturation magnetization and coercivity, followed by iron, nickel, and zinc ferrite. Furthermore, PMA-coated nanoparticles exhibit a small change in saturation magnetization, which is still enough to manipulate NPs using an external magnetic field [28].

**Table 3:** Analysis of magnetic parameters for MFe_2_O_4_ (M = Fe, Co, Ni, Zn) nanoparticles.

Samples	Saturation magnetization *M*_S_ (emu/g)	Remanence value *M*_R_ (emu/g)	Coercivity *H*_c_ (Oe)

FeO	34	6.2	35
CFO	56	22	883
NFO	39	0.32	10
ZFO	25	9.21	179

The uniform size distribution of MFe_2_O_4_ (M = Fe, Co, Zn, Ni) NPs was confirmed by agarose gel electrophoresis. All samples moved towards the positive potential due to the negatively charged PMA coating (Figure 2d).

The colloidal stability (i.e., hydrodynamic size, surface charge, and polydispersity index, PDI) of NPs was assessed using dynamic light scattering (DLS). All NPs (PMA-coated, and drug-attached) dispersed in deionized water, sodium borate buffer (SBB) pH 9.0, phosphate buffer saline (PBS) pH 7.4, and DMEM were used for zeta potential measurements. The purpose of using buffers (i.e., SBB, PBS) was to get indirect surface charge information. Deionized water was used to check the influence of electrolytes on the stability of NPs [29], and DMEM was used as a representative of biological assays. All NPs (MFe_2_O_4_-PMA, MFe_2_O_4_+DOX, and MFe_2_O_4_+MTX) indicated high zeta potential values (−35 to −26 mV) in all dispersion media except DMEM (−17 to −10 mV) as shown in Table 4. The reason behind lower zeta potential values is the interaction between NPs and serum proteins present in DMEM [30]. In cell culture media, NPs agglomerate with serum proteins and are therefore recruited in cells via the protein corona effect, which increases the bioavailability of NPs by many folds [31]. PMA-coated samples have a smaller hydrodynamic size (60–93 nm) as compared to drug-loaded samples (74–110 nm), which was further increased (132–210 nm) in DMEM due to the interaction between proteins and samples. Among them, NFO has the largest hydrodynamic size (>200 nm) in DMEM, which is not considered suitable for biological applications [30]. All samples have lower PDI values (0.13–0.33) which indicates a uniform distribution of NPs in different dispersion media (Table 5).

**Table 4:** Zeta potential, and average hydrodynamic diameter (*D*_z_) values of MFe_2_O_4_ (M = Fe, Co, Zn, Ni) NPs by DLS.

Sample	Zeta potential (mV) ± SD				Hydrodynamic size (nm) ± SD			

	Water	SBB	PBS	DMEM	Water	SBB	PBS	DMEM

FeO-PMA	−33 ± 1.1	−31 ± 1.3	−26 ± 1.9	−17 ± 2.1	46 ± 3	66 ± 4	64 ± 4	132 ± 5
FeO+DOX	−29 ± 1.7	−25 ± 1.4	−25 ± 1.1	−15 ± 1.8	85 ± 3	84 ± 5	110 ± 2	152 ± 8
FeO+MTX	−33 ± 1.6	−31 ± 1.9	−27 ± 1.9	−16 ± 1.9	91 ± 3	89 ± 5	116 ± 3	157 ± 6
CFO-PMA	−35 ± 1.8	−27 ± 1.5	−31 ± 1.6	−17 ± 1.3	46 ± 3	62 ± 2	70 ± 5	86 ± 3
CFO+DOX	−32 ± 1.1	−29 ± 1.6	−27 ± 0.9	−15 ± 1.7	74 ± 6	83 ± 5	92 ± 4	117 ± 7
CFO+MTX	−31 ± 1.3	−26 ± 1.2	−26 ± 1.6	−14 ± 1.6	64 ± 2	89 ± 7	99 ± 3	145 ± 5
NFO-PMA	−25 ± 1.1	−23 ± 1	−27 ± 0.7	−13 ± 1.2	84 ± 6	92 ± 3	160 ± 4	151 ± 8
NFO+DOX	−24 ± 1.2	−21 ± 2.1	−23 ± 2.6	−10 ± 1.3	104 ± 6	108 ± 3	163 ± 7	203 ± 5
NFO+MTX	−21 ± 1.1	−20 ± 2.3	−24 ± 1.9	−11 ± 1.5	110 ± 6	129 ± 5	157 ± 6	210 ± 3
ZFO-PMA	−34 ± 1.6	−29 ± 1.9	−29 ± 1.3	−17 ± 2.3	54 ± 5	65 ± 4	93 ± 1	135 ± 6
ZFO+DOX	−30 ± 1.3	−29 ± 2.2	−28 ± 1.9	−16 ± 1.9	74 ± 3	86 ± 5	121 ± 4	153 ± 6
ZFO+MTX	−31 ± 1.7	−27 ± 1.5	−27 ± 1.5	−13 ± 1.8	79 ± 4	93 ± 4	110 ± 3	157 ± 8

**Table 5:** PDI values of MFe_2_O_4_ (M = Fe, Co, Zn, Ni) NPs by DLS.

Sample	Water	PBS	SBB	DMEM

FeO-PMA	0.21	0.29	0.20	0.37
FeO+DOX	0.27	0.25	0.21	0.45
FeO+MTX	0.24	0.27	0.29	0.46
CFO-PMA	0.13	0.17	0.27	0.37
CFO+DOX	0.19	0.21	0.29	0.49
CFO+MTX	0.18	0.20	0.31	0.46
NFO-PMA	0.26	0.35	0.33	0.41
NFO+DOX	0.33	0.41	0.41	0.51
NFO+MTX	0.31	0.39	0.42	0.67
ZFO-PMA	0.18	0.15	0.20	0.39
ZFO+DOX	0.19	0.17	0.27	0.41
ZFO+MTX	0.19	0.21	0.29	0.46

### pH-dependent drug-loading and drug-release kinetics

**Figure 3:**
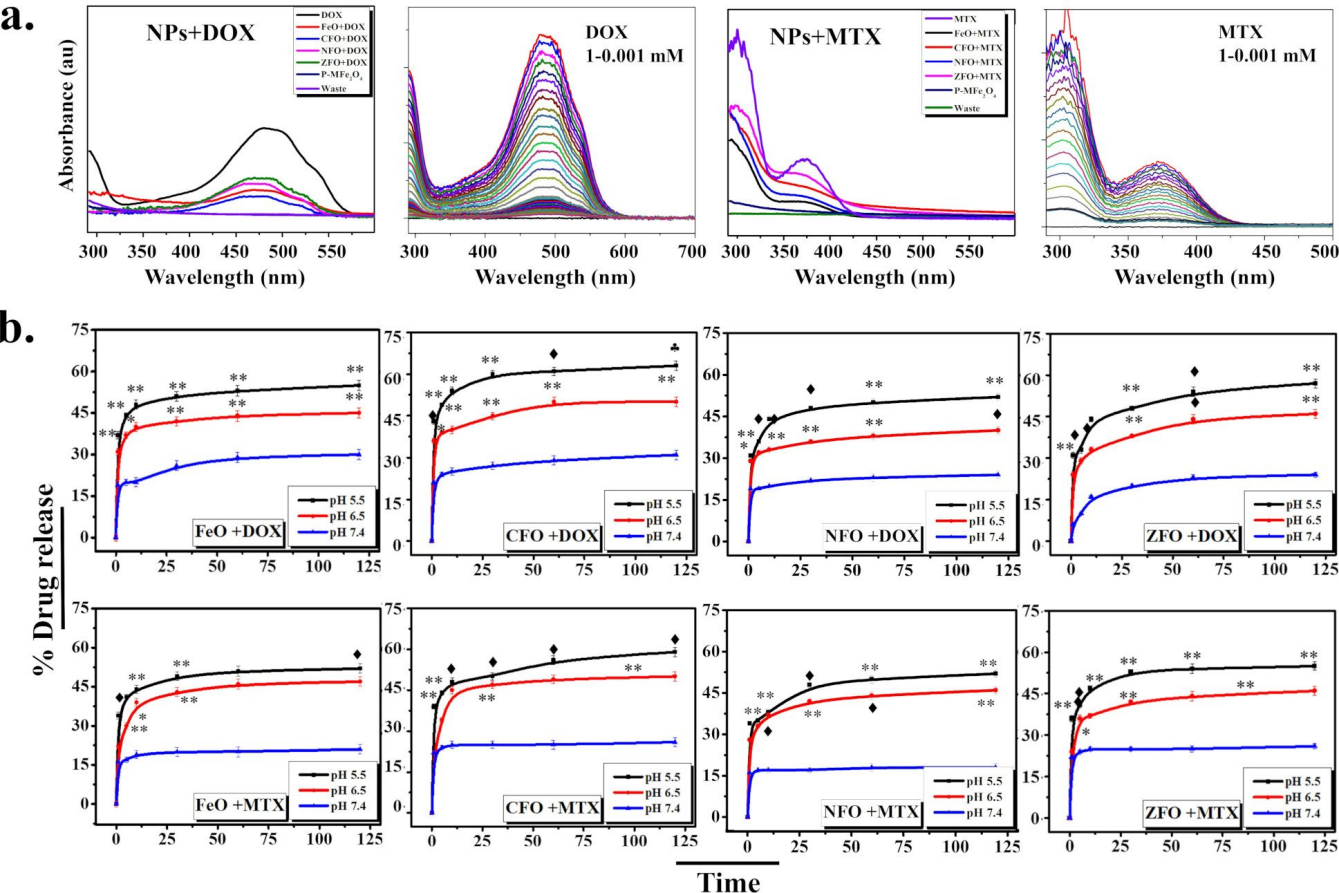
(a) UV–vis absorbance plot of DOX- and MTX-loaded MFe_2_O_4_ (M = Fe, Co, Zn, Ni) NPs indicating drug attachment along with drug titration curves. (b) Drug release kinetics of DOX- and MTX-loaded NPs at pH 5.5, 6.5, and 7.4 at different time intervals (0–120 min). The results indicate mean ± SD of three independent experiments. *p* < 0.05 (one asterisk), *p* < 0.005 (double asterisk), *p* < 0.01 (black club symbol), and *p* < 0.001 (black diamond symbol), paired two-tailed *t*-test when compared to pH 7.4.

The UV–vis-based confirmation of drug (DOX and MTX) attachment to PMA-coated MFe_2_O_4_ (M = Fe, Co, Zn, Ni) NPs is shown in Figure 3a. Samples were washed and concentrated with centrifugal filters many times to remove unattached drugs. Attached DOX and MTX were indicated at 480 and 372 nm, respectively. The samples NPs-PMA, drug only, and centrifugal filter wastes were also included for comparison. We used 0.5 mM of drugs for the loading on NPs. The encapsulated and loaded drug % for DOX and MTX are given in Table 6.

**Table 6:** Encapsulation efficiency (EE) and loading capacity (LC) of MFe_2_O_4_ (M = Fe, Co, Ni, Zn) nanoparticles.

Nanoparticles	EE% DOX	EE (μM) at 5 μg/mL	EE% MTX	EE (μM) at 5 μg/mL	LC% DOX	LC% MTX

FeO	79	0.08	83	0.08	39	41
CFO	84	0.21	82	0.21	42	46
NFO	78	0.09	80	0.10	37	40
ZFO	79	0.09	82	0.10	43	45

For pH-dependent drug-release kinetics, drug-loaded NPs were dispersed in solutions with different pH values (1× PBS; pH 5.5, 6.5, and 7.4) at room temperature and the amount of release (%) was investigated over a time interval (0–120 min). A strong pH-dependent drug release was observed at a lower pH value of 5.5 (*p* < 0.005) in all samples. For drug-loaded NPs, a burst in drug release was observed within the initial 5–10 min (Figure 3b) which indicates that the amide bonds between the drug molecules and NPs were acid labile in nature, resulting in the detachment of the drug from NPs under acidic conditions (pH. 5.5) [32]. The drug release became slower and sustained after that. Acidic conditions change the surface charge density which causes deionization of amide bonds, resulting in drug release [33–34]. From MFe_2_O_4_ (M = Fe, Co, Zn, Ni) NPs, CFO had the highest total drug release for DOX and MTX (percent release = 62 ± 0.99 and 59 ± 1.19, respectively) at a lower pH value (5.5), followed by ZFO, FeO, and NFO as shown in Table 7. The drug release behavior at pH 7.4, 6.5, and 5.5 for DOX and MTX shows an increasing curve from higher (7.4) to lower (5.5) pH values. A small amount of release (20–30%) for DOX and MTX was observed at pH 7.4, which indicates that the pH-dependent release behavior may contribute to efficient drug delivery at tumor sites where an acidic microenvironment is prevalent [35], with lesser premature drug release in circulation and in normal cells, where the pH value is maintained at 7.4. Furthermore, once NPs are internalized by tumor cells, the acidic environment in the endosome may also trigger hydrolysis of amide bonds present between the drug and the polymer, thereby rapidly releasing the drug from NPs into the cytosol [36].

**Table 7:** Total drug (DOX/MTX) release from MFe_2_O_4_ (M = Fe, Co, Zn, Ni) nanoparticles, at pH 5.5, 6.5, and 7.4 after 120 min.

Nanoparticles	DOX			MTX		

	pH 5.5	pH 6.5	pH 7.4	pH 5.5	pH 6.5	pH 7.4

FeO	54 ± 1.05	45 ± 1.23	30 ± 0.89	52 ± 1.15	46 ± 1.11	23 ± 0.87
CFO	62 ± 0.99	49 ± 1.11	29 ± 1.09	59 ± 1.19	49 ± 0.91	26 ± 1.12
NFO	51 ± 1.02	40 ± 0.90	25 ± 0.92	52 ± 1.08	43 ± 1.07	19 ± 0.91
ZFO	57 ± 1.30	46 ± 1.22	25 ± 1.23	54 ± 1.16	45 ± 0.99	25 ± 0.92

### Functionalized MFe_2_O_4_ NPs cause cytotoxicity in vitro

The sulforhodamine B (SRB) assay was performed using HepG2 and HT144 cells to screen the cytotoxic potential of functionalized MFe_2_O_4_ (M = Fe, Co, Ni, Zn) NPs. The cells were exposed to 5 μg/mL of NPs for 24 h. For a better comparison, free drug (DOX and MTX) controls were included, which were equivalent to the total drug amount attached (with a given dose of NPs as mentioned in Table 6). PMA-coated NPs (5 μg/mL) and untreated cultures were also included as controls.

**Figure 4:**
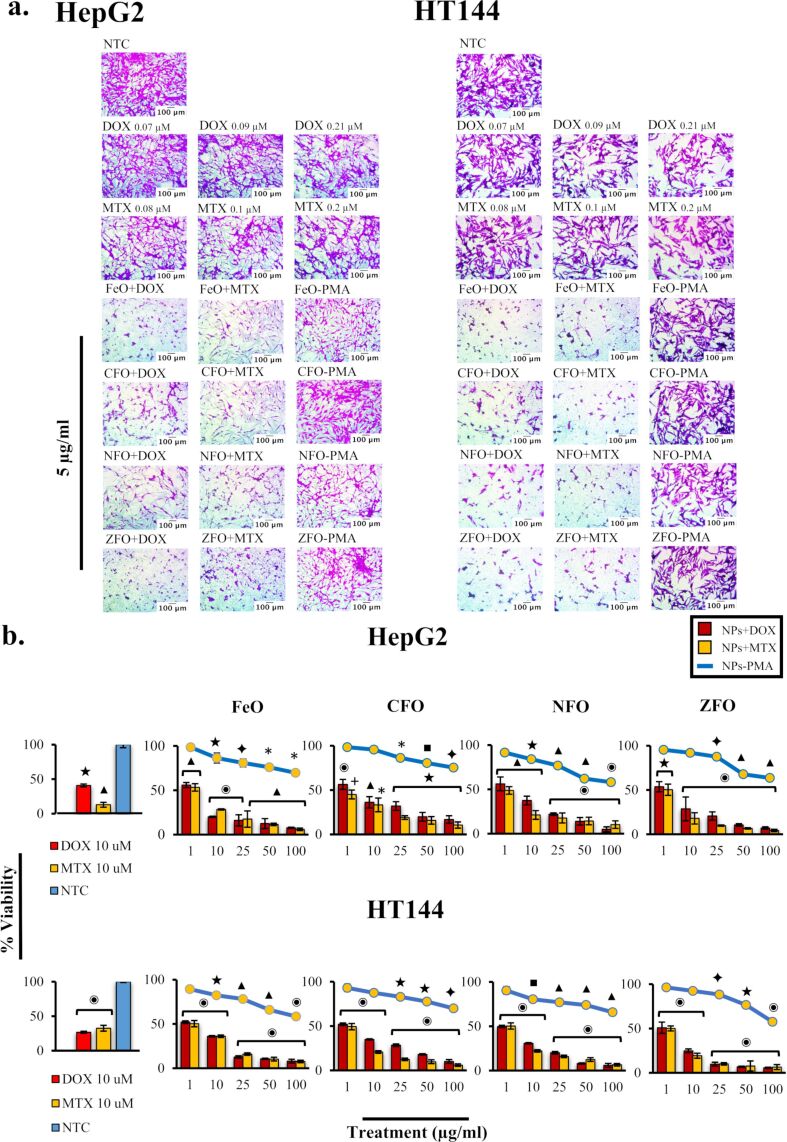
(a) Microscopy images of HepG2 and HT144 cells treated with drug-loaded (DOX and MTX) MFe_2_O_4_ (M = Fe, Co, Ni, Zn) NPs at a 5 μg/mL dose, for 24 h. For comparison, free drug controls were included, which represented the total drug amount attached to NPs at different dosages (DOX = 0.08, 0.21, 0.09, and 0.09 μM; MTX = 0.08, 0.2, 0.1, and 0.1 μM respectively). Nontreated controls and PMA-coated samples (NPs-PMA) were also included as controls (5 μg/mL). Magnification = 200×, scale bar = 100 μm. (b) Dose-dependent cytotoxicity of HepG2 and HT144 cells treated with NPs+drugs and NPs-PMA (1, 10, 25, 50, and 100 µg/mL) for 24 h. Controls included free DOX and MTX (10 μM each) and NTC. Plotted data indicates mean ± SD of independent triplicates. *p* < 0.05 (one asterisk), *p* < 0.01 (black square symbol), *p* < 0.005 (black diamond symbol), *p* < 0.001 (black star symbol), *p* < 0.0005 (black triangle symbol), and *p* < 0.0001(circled bullet symbol), paired two-tailed *t*-test when samples were compared to NTC.

The efficient retention of polymer-functionalized NPs in cancer cells, with the help of the EPR effect and a leaky vasculature system (pore diameter = 100 nm–2 µm) [7], reduces their nonspecific biological interactions with plasma proteins, contributing to a higher bioavailability [37]. SRB screening results for HepG2 and HT144 (Figure 4a) cells showed a strong cytotoxic effect (% viability < 50%) upon treatment with drug-loaded NPs compared to nontreated cells (NTC). This cytotoxic effect was prominent when compared to free drug controls, where cell viability was up to 70–80%, indicating higher bioavailability and better internalization of anticancer drugs when loaded on ferrite NPs.

Treated cells also exhibited morphological alterations such as cellular shrinkage and elongation which may affect their ability to metastasize (i.e., to adhere, migrate, and invade) [38]. PMA-coated NPs showed viability up to 80%, indicating excellent biocompatibility of amphiphilic polymers at a lower dose in vitro*.*

### IC_50_ concentrations of functionalized MFe_2_O_4_ NPs in cancer cells

To determine IC_50_ concentrations of drug-loaded NPs and their effect on the metabolic activity of HepG2 and HT144 cells, MTT (3-(4,5-dimethylthiazol-2-yl)-2,5-diphenyltetrazolium bromide) assays were performed. Cells were exposed to several concentrations (1, 10, 25, 50, and 100 µg/mL) of NPs+drugs and NPs-PMA for 24 h. Nontreated samples and free drugs (DOX and MTX, 10 μM each) were included as controls. Percentage values of cell viability were plotted for all doses (Figure 4b) and IC_50_ values were determined (Table 8). In both cell lines, drug-functionalized NPs caused almost 45–50% reduction in cellular viability at a concentration of 1 µg/mL, and the cellular viability decreased even further at higher doses.

**Table 8:** IC_50_ values (μg/mL) of drug-loaded MFe_2_O_4_ nanoparticles with attached drugs (μM).

Sample	HepG2		HT144		Lymphocytes	

	IC_50_ (μg/mL)	Attached drug (μM)	IC_50_ (μg/mL)	Attached drug (μM)	IC_50_ (μg/mL)	Attached drug (μM)

FeO+DOX	2.48	0.04	2.18	0.03	22.68	0.36
CFO+DOX	3.81	0.16	2.08	0.09	35.96	1.51
NFO+DOX	3.97	0.07	0.86	0.01	18.35	0.32
ZFO+DOX	2.34	0.07	1.30	0.02	24.54	0.73
FeO+MTX	2.18	0.03	1.18	0.01	41.65	0.57
CFO+MTX	1.23	0.05	0.81	0.03	43.04	1.75
NFO+MTX	2.31	0.02	1.09	0.02	21.04	0.18
ZFO+MTX	1.08	0.02	1.02	0.02	38.71	0.71

In HepG2 cells, cellular viability at a dose of 1 µg/mL ranged from 53.72 ± 5.65% to 56.40 ± 5.46% (*p* < 0.001) for NPs+DOX and 50.30 ± 4.94 to 54.51 ± 4.24% (*p* < 0.05) for MTX-loaded NPs. ZFO NPs were the most cytotoxic ones (IC_50_ = 2.34 µg/mL and 1.08 µg/mL for ZFO+DOX and ZFO+MTX, respectively). In HT144 cells, cellular viability values of 49.70 ± 1.41 to 52.10 ± 1.45% and 49.40 ± 3.53 to 50.29 ± 3.21 (*p* < 0.0001) were observed upon treatments with NPs+DOX and NPs+MTX (1 µg/mL), respectively. NFO+DOX (IC_50_ = 0.86 µg/mL) and CFO+MTX (IC_50_ = 0.81 µg/mL) were the most cytotoxic ones.

PMA-coated NPs used as a control in this study were comparatively nontoxic to the cells with a relative percentage viability ranging from 98.53 ± 0.76% to 84.11 ± 1.29% against HepG2 using different NPs-PMA at various concentrations. Similarly, for HT144 the percentage viability ranged from 96.35 ± 0.50 to 80.4 ± 2.48%, indicating biocompatibility and higher tolerance of the drug-free particles.

The drug-loaded NPs showed 1.6- to 12-fold stronger effects when compared to the NPs-PMA at the same doses.

### Functionalized MFe_2_O_4_ NPs cause apoptosis in a dose-dependent manner

**Figure 5:**
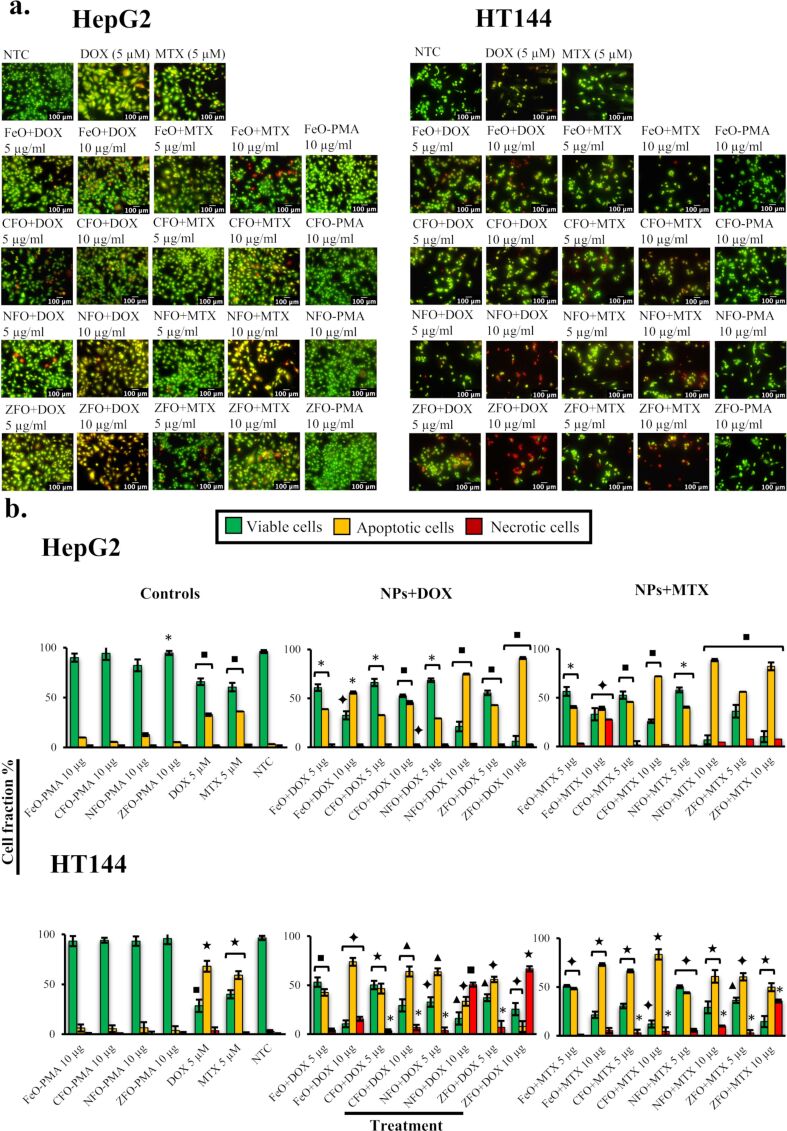
(a) Fluorescence microscopy images of HepG2 and HT144 cells upon treatment with drug-loaded (DOX and MTX) MFe_2_O_4_ (M = Fe, Co, Ni, Zn) NPs at 5 and 10 μg/mL for 3 h. Controls included free drugs (DOX and MTX, 5 μM), NPs-PMA (10 μg/mL), and NTC. Live cells are shown in green, necrotic cells are shown in red, and apoptotic cells are shown in yellow to orange range due to AOPI staining. Magnification = 200×, scale bar = 100 μm. (b) Quantitative analysis of the percentage of viable, apoptotic, and necrotic cells (mean ± SD of experimental triplicates) in treated HepG2 and HT144 cells in comparison to controls. *p* < 0.05 (one asterisk), *p* < 0.01 (black square symbol), *p* < 0.005 (black diamond symbol), *p* < 0.001 (black star symbol), and *p* < 0.0005 (black triangle symbol), two-tailed *t*-test when samples were compared to NTC.

Fractions of live, dead, and apoptotic cells (HepG2 and HT144) were quantitatively determined by fluorescence microscopy using acridine orange and propidium iodide (AOPI) staining. Cells were treated with drug-loaded NPs for 3 h at concentration values of 5 and 10 μg/mL. NTC, NPs-PMA (10 μg/mL) and free drugs (DOX and MTX, 5 μM each) were included as controls. After treatment and staining with AOPI, cells were observed under a fluorescence microscope where viable cells appeared green, apoptotic cells appeared orange/yellow, and necrotic cells appeared red in color (Figure 5a). Percentage fractions of live, necrotic, and apoptotic cells were calculated in each replicate and compared to NTC (Figure 5b). Spinel ferrite NPs have been reported to cause cytotoxicity in cancer cells, mediated via ROS production, increased transcription of p53 and apoptotic genes (caspase 3 and 9, Bax), and downregulation of the antiapoptotic Bcl-2 gene, leading to programmed cell death [39]. However, the chemical composition of NPs plays an important role in determining cytotoxic behavior. For example, NPs with cores made up with toxic metals (e.g., Cd and Ag) cause cytotoxicity. Conversely, metals such as Fe and Zn are biocompatible and cause toxicity at higher doses or by leakage of the metal ions from the core surface, causing oxidative stress. This phenomenon can be controlled by coating the NPs with polymeric shells, which enhances their biocompatibility and stability [40].

All drug-loaded samples exhibited a dose-dependent response. Among DOX-loaded NPs, ZFO+DOX was the most cytotoxic in HepG2 cells, with an apoptotic cell fraction of 43.12 ± 2.35% at a 5 µg/mL dose (*p* < 0.01). Conversely, FeO+DOX, CFO+DOX, and NFO+DOX showed 39 ± 3.39, 32.83 ± 3.81, and 29.5 ± 1.93% of apoptotic cells (*p* < 0.05), respectively. The percentage of apoptotic cells increased by 1.4- to 2.5-fold (*p* < 0.05) at higher doses, with maximum apoptosis observed in ZFO+DOX (91.24 ± 5.43%). In case of MTX-loaded samples, approximately 40.51 ± 3.70 to 56.26 ± 5.34% of apoptotic cells (*p* < 0.05) were observed at a 5 µg/mL dose, with ZFO+MTX being the most cytotoxic. The apoptotic fraction increased up to 2-fold in the presence of CFO+MTX, NFO+MTX, and ZFO+MTX at a 10 µg/mL dose (*p* < 0.01), whereas FeO+MTX exhibited a 9-fold increase in necrotic cells (*p* < 0.005). Free drugs (DOX and MTX, 5 μM each) used as controls resulted in 33.07 ± 3.72 and 36.25 ± 3.23% of apoptotic cells (*p* < 0.01), respectively.

In HT144 cells, 42.15 ± 3.45 to 63.62 ± 3.51% of apoptotic cells (*p* < 0.05) were observed in the presence of DOX-loaded NPs (5 μg/mL) with the highest apoptotic fraction of 63.66 ± 3.5% upon NFO+DOX treatment. An increase in apoptotic cells up to 1.3- and 1.7-fold (*p* < 0.01) was observed in CFO and FeO+DOX, respectively, at a 10 µg/mL dose. Alternatively, a significantly (*p* < 0.01) high cell death (9.6- to 14-fold increase in necrotic cells) was observed in ZFO and NFO+DOX nanocarriers, respectively. In the presence of MTX-loaded NPs, CFO and ZFO showed the maximum amount of apoptotic cells (i.e., 66.57 ± 1.39 and 60.52 ± 3.81% respectively, *p* < 0.005) at a 5 μg/mL concentration. At a higher dose, the percent of apoptotic cells increased up to 1.5-fold in FeO, CFO, and NFO+MTX (*p* < 0.005). ZFO+MTX, however, caused maximum cell death with a 12-fold increase in necrotic cells. DOX and MTX controls caused 68.05 ± 5.55 and 59.13 ± 3.93% of apoptosis, respectively (*p* < 0.001).

In both cell lines, PMA-coated NPs showed higher cellular viability after 3 h of treatment at a 10 µg/mL dose (HepG2 = 82.22 ± 5.92 to 94.74 ± 2.03%; HT144 = 93.36 ± 5.11 to 95.91 ± 5.73%), indicating biocompatibility of NPs-PMA at a given dose and treatment time.

### Cells undergo oxidative stress upon treatment with functionalized MFe_2_O_4_ NPs

Generation of ROS has been associated with DNA damage, inflammation, apoptosis and senescence in cells [41]. The 2',7'-dichlorodihydrofluorescein diacetate (H_2_-DCFDA) assay was used to determine cellular ROS production in HepG2 and HT144 cells upon treatment with NPs over a period of time. Cells were exposed to NPs+DOX and NPs+MTX at IC_50_ concentrations and the increase in fluorescence was determined relative to untreated control cells over a time interval (0–45 min). Several studies have indicated that spinel ferrite MFe_2_O_4_ (M = Fe, Co, Ni, Zn) NPs cause cytotoxicity via oxidative stress which results in damage to the cell membrane, proteins, and DNA [41–43]. However, how NPs are processed inside the cell is also a contributing factor in ROS production [44]. For example, metallic NPs transported to lysosomes produce more ROS due to enhanced acidic degradation as compared to those remaining in the cytosol [45]. Besides, the intrinsic antioxidant potential of various cells also contributes to biocompatibility and extent of ROS generation via metallic NPs [46–47].

**Figure 6:**
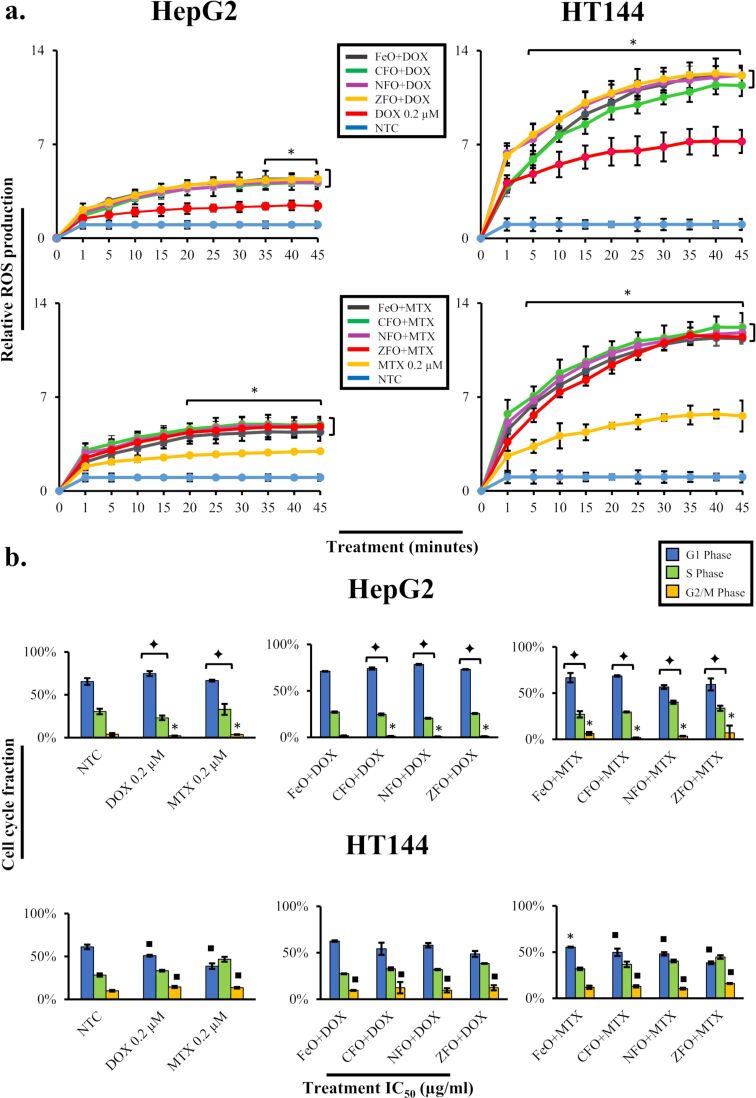
(a) Intracellular ROS generation in HepG2 and HT144 cells upon treatment with drug-loaded (DOX and MTX) MFe_2_O_4_ (M = Fe, Co, Ni, Zn) NPs for 0–45 min at IC_50_ doses. Free drugs (DOX and MTX, 0.2 µM each) and NTC were included as controls. ROS generation in cells was determined relative to NTC. Data was represented as mean ± SD of experimental triplicates. *p* < 0.05 (one asterisk), paired two-tailed *t*-test when compared to NTC. (b) Effect of drug-loaded MFe_2_O_4_ NPs on cell cycle progression in HepG2 and HT144 cells determined by flow cytometry. Cells were exposed to IC_50_ doses of NPs for 24 h. Untreated cells and free drugs (DOX and MTX, 0.2 µM) were included as controls. *p* < 0.05 (one asterisk), *p* < 0.01 (black square symbol), *p* < 0.005 (black diamond symbol), paired two-tailed *t*-test when compared to NTC.

In the present work, free DOX and MTX produced non-significant ROS in both cell lines, which significantly enhanced upon NP-mediated drug delivery. An increase in ROS production was observed in the first 5 min of treatment and steadily increased over time. NPs+DOX produced a significant amount of ROS (*p* < 0.05) in HepG2 cells (Figure 6a) after 35–45 min of treatment. Conversely, NPs+MTX showed significant (*p* < 0.05) results after 20–45 min of exposure. Drug-loaded NPs exhibited 1.6- to 2-fold ROS production in HepG2 cells compared to free drug controls. HT144 cells, however, were more susceptible to oxidative damage via NPs+drugs and free drug controls compared to HepG2 cells. Both NPs+DOX and NPs+MTX started to produce a significant (*p* < 0.05) ROS amount at 5 min exposure time with 2- to 3-fold increased effect compared to free drug controls. Lower sensitivity of HepG2 cells towards oxidative stress can be attributed to the presence of xenobiotic detoxification and antioxidant mechanisms [47].

### Functionalized MFe_2_O_4_ NPs cause cell cycle arrest in cancer cells

Evidence of oxidative damage in treated cells may indicate DNA damage and possible effects on cell cycle progression, causing damaged cells to accumulate in sub-G1, G1, S, or G2/M phases of the cell cycle [48]. To determine the effects of drug-loaded MFe_2_O_4_ NPs on the cell cycle progression, HepG2 and HT144 cells treated with NPs at IC_50_ doses were analyzed by flow cytometry. The data obtained from each sample was compared to NTC (Figure 6b).

In HepG2 cells, all NPs+DOX, FeO+MTX, and CFO+MTX showed a significant (*p* < 0.005) G1 arrest resulting in a decreased cellular population in S and G2/M phases (*p* < 0.05). Similar observations were made in free DOX (0.2 µM) control, which was consistent with a previous report [49]. In contrast, NFO+MTX and ZFO+MTX exhibited a delay in the S phase (*p* < 0.005) causing inhibition of cellular replication and progression towards G2/M. Furthermore, a significant (*p* < 0.05) G2/M phase arrest in FeO+MTX and ZFO+MTX was also observed. Free MTX (0.2 µM) control caused G1 and S phase arrest (*p* < 0.005) with reduced cells in the G2/M phase (*p* < 0.05). Methotrexate has been previously reported to cause cytotoxicity in the S phase and stop progression from G1 to S phase [50].

In HT144 cells, all DOX-loaded NPs showed a non-significant S phase arrest except FeO+DOX which caused G1 arrest. CFO+DOX and ZFO+DOX were also responsible for G2/M arrest (*p* < 0.01) accompanied by a lower cell population at G1. Comparably, free DOX control also caused a G2/M arrest (*p* < 0.01) and reduced the number of cells in the G1 phase (*p* < 0.01). All MTX-loaded NPs and free MTX control caused G2/M arrest (*p* < 0.01) in cells with a non-significant S phase arrest, lowering the cellular population in the G1 phase (*p* < 0.01).

The obtained flow cytometry results from drug-loaded NPs were comparable to free drug controls (0.2 µM). However, considering that the amount of attached drug with IC_50_ doses of NPs was much lower than 0.2 µM (as mentioned in Table 8), it was speculated that nanocarriers amplified the effects due to improved and efficient drug delivery at a much lower dose compared to free drug controls.

### Functionalized MFe_2_O_4_ NPs cause genotoxicity in treated cells

**Figure 7:**
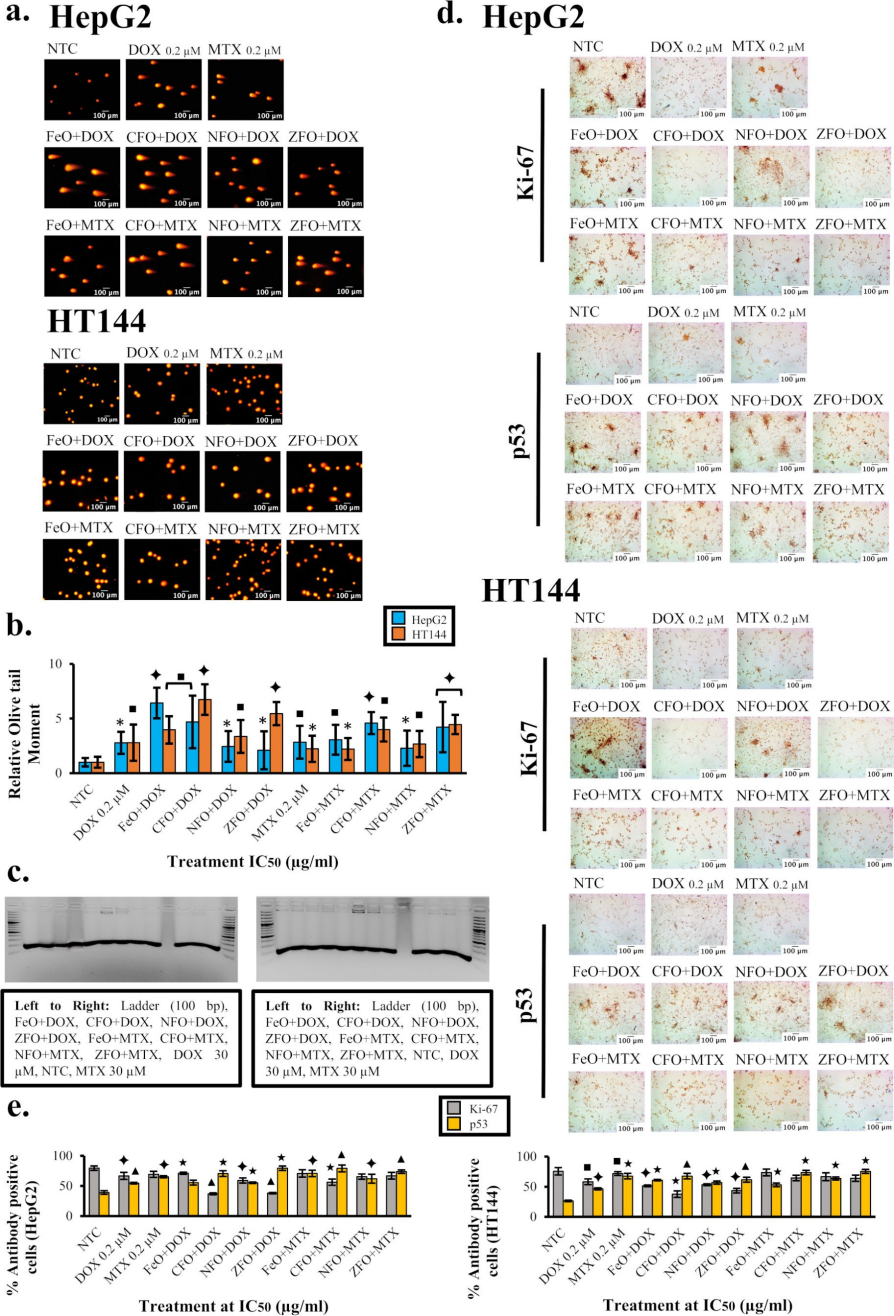
(a) Comet images of HepG2 and HT144 cells treated with drug-loaded (DOX and MTX) MFe_2_O_4_ (M = Fe, Co, Ni, Zn) NPs at IC_50_ doses for 1 h. Controls included free drugs (DOX and MTX, 0.2 μM each) and NTC. Cells were stained with propidium iodide (5 µg/mL). Magnification = 200×, scale bar = 100 μm. (b) Olive tail moment values as a measurement of DNA damage (mean ± SD) were calculated relative to NTC for each sample. *p* < 0.05 (one asterisk), *p* < 0.01 (black square symbol), and *p* < 0.005 (black diamond symbol), paired two-tailed *t*-test when compared to NTC. (c) From left to right: apoptotic DNA fragmentation in HepG2 and HT144 cells after treatment with NPs+drugs for 24 h at IC_50_ doses. Free drugs (DOX and MTX, 30 µM) and NTC were included as controls. DNA samples were electrophoresed on a 2% agarose gel for 2 h at 50 V along with a DNA ladder (100 bp). (d) Microscopy images of immunocytochemical (ICC) staining of treated (IC_50_ doses of NPs+drugs for 24 h) HepG2 and HT144 cells with Ki-67 and p53 mouse monoclonal antibodies. Controls included NTC and free drugs (DOX and MTX, 0.2 μM). Magnification = 200×, scale bar = 100 μm. (e) Bar charts representing the expression levels of Ki-67 and p53 antibodies in treated HepG2 and HT144 cells. Antibody positive cells were counted in treated and untreated samples and percentages were calculated (mean ± SD). *p* < 0.01 (black square symbol), *p* < 0.005 (black diamond symbol), *p* < 0.001 (black star symbol), and *p* < 0.0005 (black triangle symbol), paired two-tailed *t*-test upon comparison with NTC.

The alkaline comet assay was performed to determine the genotoxicity of drug-loaded NPs in HepG2 and HT144 cells (Figure 7a). The olive tail moment was measured for each sample and a relative calculation with respect to NTC was performed as a measurement of DNA damage (Figure 7b). The genotoxic effect of NPs may arise from their direct interaction with DNA or enhanced ROS production by cellular components. If unrepaired or misrepaired, these lesions may contribute to replication errors and gene or chromosomal alterations [51].

The genotoxicity of spinel ferrite NPs has been reported earlier [52–53]. However, other factors such as NP size and ligand used for functionalization may contribute to a genotoxic activity. Smaller NPs (<50 nm) have large surface area which increases their biological interactions and hence genotoxic potential [54]. Polymeric coatings, on the other hand, are aimed to enhance colloidal stability and facilitate the interaction between NPs a plasma membrane associated proteins [55]. In addition, the charge of polymeric coatings also governs NP uptake by cells. Positively charged polymers have been reported to enhance genotoxicity due to better internalization of NPs via the plasma membrane (electrostatic interaction) and direct interaction with the nucleus and DNA. On the other hand, negatively charged coating such as PMA has no effect on genotoxicity [56].

In HepG2 cells, FeO+DOX and CFO+DOX showed maximum genotoxicity with relative tail moment values of 6.41 ± 1.75 and 4.68 ± 2.45 (*p* < 0.01), respectively, which were higher (up to 2-fold) than that of free DOX control (2.77 ± 2.35, *p* < 0.05). Among MTX nanocarriers, CFO+MTX and ZFO+MTX were highly genotoxic (*p* < 0.005) having relative tail moment values of 4.58 ± 4.23 and 4.21 ± 4.93, respectively. The results were up to 1.6-fold higher than that of free MTX control (2.82 ± 3.24; *p* < 0.01).

In HT144 cells, maximum genotoxicity (*p* < 0.005) was observed in CFO+DOX and ZFO+DOX with relative tail moment values of 6.72 ± 1.44 and 5.44 ± 1.06, respectively. The results were enhanced up to 2.4-fold compared to free DOX, where a relative tail moment of 2.78 ± 1.66 (*p* < 0.01) was observed. Relative tail moment values in CFO+MTX and ZFO+MTX were 3.98 ± 1.09 and 4.44 ± 0.88 (*p* < 0.005), respectively, which were almost 2-fold higher than that of free MTX control (2.22 ± 1.21; *p* < 0.05). The obtained results were therefore indicative of enhanced genotoxic behavior in cancer cells upon NP-mediated drug delivery.

### Functionalized MFe_2_O_4_ NPs cause DNA fragmentation in treated cells

Both HepG2 and HT144 cells showed apoptotic DNA fragmentation (Figure 7c) upon treatment with drug-loaded NPs at IC_50_ doses for 24 h. Distinct bands of 180 bp were visualized on a 2% agarose gel, which indicates shearing of DNA as a result of apoptosis in treated cells. These findings indicated that drug-loaded NPs cause cytotoxicity in cancer cells via oxidative stress leading to apoptosis and DNA fragmentation.

### Functionalized MFe_2_O_4_ NPs alter Ki-67 and p53 expression in treated cells

p53 is a tumor suppressor protein also known as the “guardian of the genome”. It is involved in downstream regulation of genes involved in apoptosis, DNA repair, and cell cycle arrest. Overexpression of p53 is triggered by stress stimuli, such as hypoxia, ROS, ionizing radiations, and carcinogens. Normal cells have a low expression of p53 but its half-life may increase up to several hours under stress, resulting in elevated expression [57]. Enhanced expression of p53 in response to cellular stress has been associated with cell cycle regulation. It causes G1 arrest by inhibiting cyclin D and stimulating p21 expression. It is also involved in repairing lethal DNA damages (double-stranded breaks) via Gadd45 by arresting cells at G1. The G2 arrest occurs by p53-mediated reduction in cyclin B1 and the S phase arrest happens by the regulation of mitotic spindle checkpoints. Extensive DNA damage, however, leads to apoptosis [58].

Conversely, Ki-67 is an important proliferative and prognostic cancer biomarker expressed in the nucleus during the cell cycle. It is important for cell division and biosynthesis of ribosomal RNA and it is variably expressed throughout the cell cycle (high in the G2/M phase and low in G1 and S phases). High expression of Ki-67 usually contributes to poor survival rates in cancer patients [59].

In this study, the effect of the treatment with drug-loaded MFe_2_O_4_ (M = Fe, Co, Ni, Zn) NPs (IC_50_ doses) on cancer biomarker expression levels was evaluated via ICC (Figure 7d). Untreated cells exhibited a high expression of Ki-67 (HepG2 = 79.62 ± 3.72%; HT144 = 75.67 ± 6.14%), indicating a high proliferative potential of cancer cells, whereas the expression of p53 was relatively low (HepG2 = 39.23 ± 2.91%; HT144 = 26.44 ± 1.25%) (Figure 7e).

Upon treatment, p53 expression was elevated which was potentially responsible for cell cycle arrest and apoptosis. In HepG2 cells, DOX-loaded CFO and ZFO nanocarriers showed a maximum p53 expression of 70.43 ± 4.82 and 79.47 ± 3.65% (*p* < 0.001), respectively. Similar results were observed in MTX-loaded NPs, where a maximum p53 expression of 79.13 ± 5.61 and 73.65 ± 2.93% (*p* < 0.0005) was observed in CFO+MTX and ZFO+MTX, respectively. An increase in expression was observed when compared to free drug controls, where only 54.61 ± 1.32% and 65.33 ± 1.71% of p53 expression was observed in DOX and MTX, respectively (*p* < 0.005). On the other hand, a decrease in Ki-67 expression was observed in treated cells, indicating a potential role in the inhibition of cellular proliferation. A significantly stronger (*p* < 0.005) decrease in Ki-67 expression was observed in CFO and ZFO nanocarriers (CFO+DOX = 36.9 ± 1.57%, ZFO+DOX = 38.1 ± 1.17%, CFO+MTX = 56.3 ± 5.23%, and ZFO+MTX = 66.9 ± 5.55%). Free drug controls, however, reduced Ki-67 expression by approx. 12% (DOX = 66.6 ± 5.82%, MTX = 69.2 ± 5.14%) compared to NTC.

Similar observations were made in HT144 cells where CFO and ZFO nanocarriers were highly effective. Among DOX nanocarriers, CFO+DOX showed the highest p53 expression of 67.66 ± 4.66% (*p* < 0.0005) and the lowest Ki-67 expression of 37.75 ± 5.31% (*p* < 0.001). Conversely, p53 and Ki-67 levels were 46.63 ± 1.65% (*p* < 0.005) and 58.27 ± 4.86% (*p* < 0.01) in the free DOX control. Amidst MTX nanocarriers, Ki-67 levels of 64.44 ± 4.62% and 64.15 ± 5.21% were observed in CFO+MTX and ZFO+MTX, respectively. Both nanocarriers also elevated p53 expression up to 75% (*p* < 0.001). Free MTX, however, showed Ki-67 and p53 levels of 71.82 ± 3.11% (*p* < 0.01) and 67.66 ± 4.68% (*p* < 0.001), respectively, indicating a better performance of drug-loaded NPs. The obtained results also suggested a stronger inhibition of Ki-67 by DOX-loaded NPs as compared to NP+MTX in both cell lines.

In both cell lines, a decrease in Ki-67 expression may indicate a low proliferative potential of cancer cells after treatment with nanocarriers. However, a variable expression of Ki-67 during the cell cycle may also affect these findings [60]. Flow cytometry results revealed that NPs+DOX caused G1 and S phase arrest in HepG2 and HT144 cells, respectively, which may result in a relatively low expression of Ki-67 in these treatment groups and a comparatively higher expression in samples showing G2/M arrest in the cell cycle. Furthermore, irreparable DNA damage (double-stranded breaks) also contributes towards irreversible G1 arrest and senescence, which decreases the proliferative capacity of the cells [61]. Since the effect of Ki-67 on cell survival and proliferation has not been clearly understood yet [59], it is not possible to elucidate the effect of certain therapeutic interventions on this biological event without extensive investigations.

### Functionalized MFe_2_O_4_ NPs reduce cellular viability in HepG2 and HT144 3D spheroid models

The use of three-dimensional (3D) spheroid models for high-throughput drug screening in vitro is favored due to their close resemblance to in vivo tumors. Moreover, they possess several tumor hallmarks, such as hypoxia, cellular interaction, drug resistance, and dense extracellular matrix, allowing for better pathobiological studies of human cancers [62].

**Figure 8:**
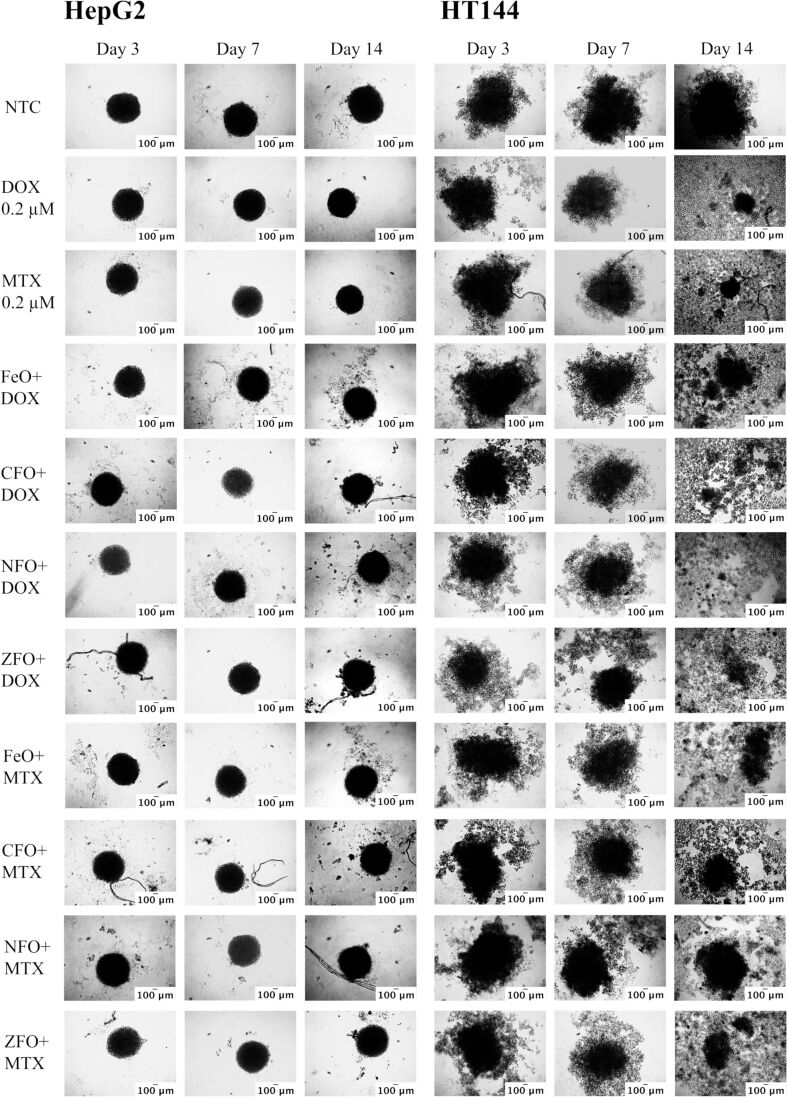
Microscopy images (magnification = 100×, scale bar = 100 µm) of HepG2 and HT144 3D spheroids grown for three days and then treated with drug-loaded (DOX and MTX) MFe_2_O_4_ (M = Fe, Co, Ni, Zn) NPs at 5 µg/mL for 14 days. Controls included free drugs (DOX and MTX, 0.2 µM each) and NTC. The medium was replenished after regular intervals and photographs were taken after three, seven, and fourteen days to observe changes in spheroid morphology.

Here, spheroids of HepG2 and HT144 cells were grown for cytotoxicity assessment of drug-functionalized MFe_2_O_4_ NPs (5 µg/mL). The obtained HepG2 and HT144 spheroids had average diameters of 420 ± 21.5 and 582 ± 72 µm, respectively, which reached the maximum value at the 14th day (HepG2 = 450 ± 16.33 and HT144 = 713 ± 81.3 µm). HepG2 formed compactly packed spheroids, whereas HT144 spheroids were loosely bound (Figure 8).

**Figure 9:**
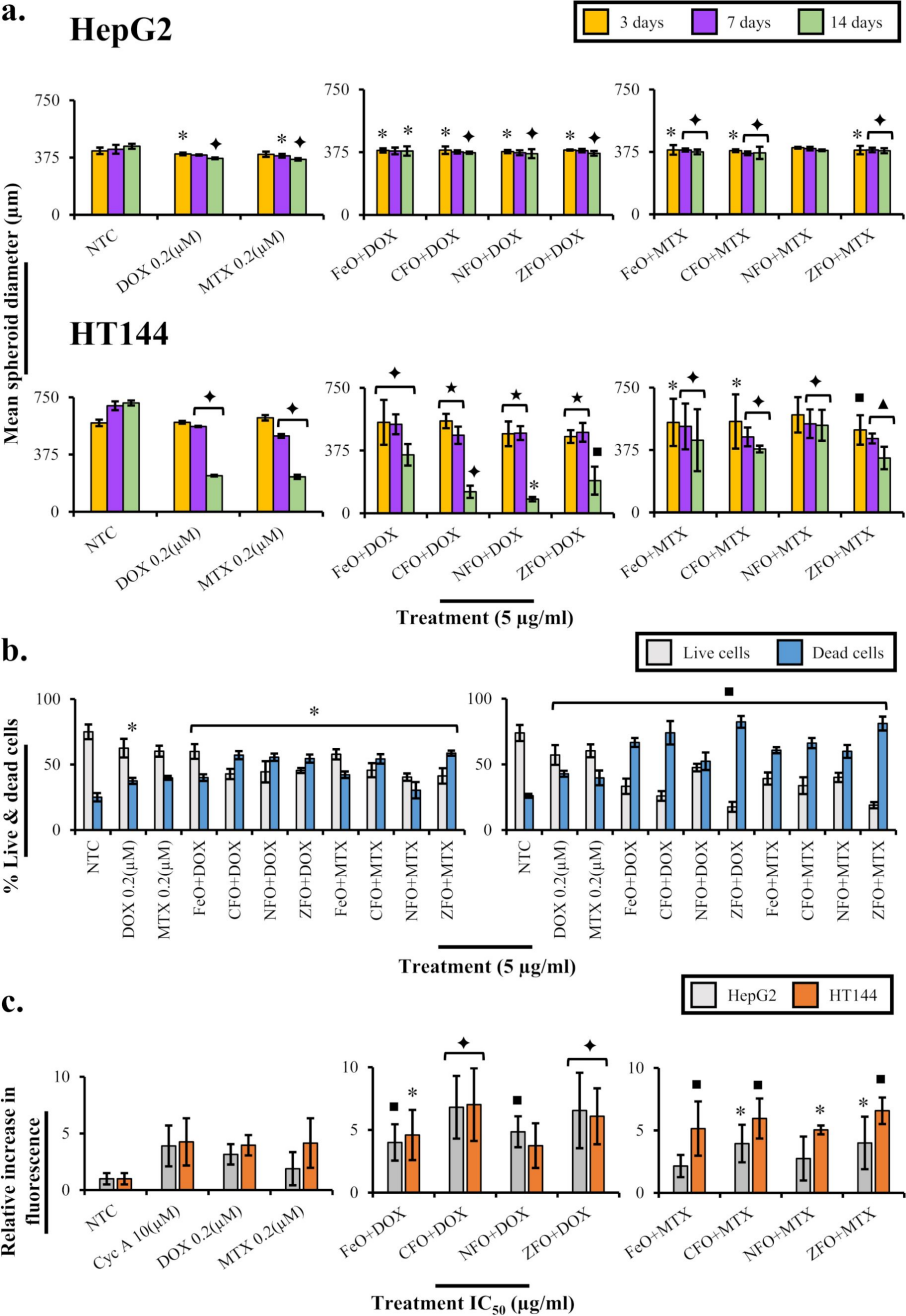
(a) Bar charts indicating changes in HepG2 and HT144 spheroid diameters upon treatment with drug-loaded (DOX and MTX) MFe_2_O_4_ (M = Fe, Co, Ni, Zn) NPs at 5 µg/mL for three, seven, and fourteen days. Controls included free drugs (DOX and MTX, 0.2 µM each) and NTC. After each time point, an average spheroid diameter was calculated by the ImageJ software. The plotted data indicates mean ± SD of multiple readings covering maximum and minimum diameter ranges of spheroids. (b) From left to right: bar charts indicating viability and death percentage in treated HepG2 and HT144 3D spheroids after 14 days. Live and dead cells in each sample were counted using the trypan blue assay. The data plotted indicates mean ± SD of three replicates. *p* < 0.05 (one asterisk), *p* < 0.01 (black square symbol), *p* < 0.005 (black diamond symbol), *p* < 0.001 (black star symbol), and *p* < 0.0005 (black triangle symbol), paired two-tailed *t*-test when compared to NTC. (c) Bar charts represent multidrug resistance (MDR) pump inhibition (mean ± SD) in HepG2 and HT144 cells treated with NPs+drugs for 24 h. Controls included NTC, free drugs (DOX and MTX, 0.2 µM each), and the MDR inhibitor cyclosporin A (Cyc A = 10 µM). The inhibition of MDR pump activity was determined by the increase in fluorescence relative to NTC. *p* < 0.05 (one asterisk), *p* < 0.01 (black square symbol), and *p* < 0.005 (black diamond symbol), paired two-tailed *t*-test when samples were compared to free drug controls.

In HepG2 spheroids, a slight reduction in the spheroid diameter was observed after treatment with drug-loaded NPs. The spheroids were also not highly disintegrated (Figure 8). ZFO+DOX and CFO+MTX, among DOX and MTX nanocarriers, caused a maximum reduction (*p* < 0.005) in spheroid diameter up to 20 µm (Figure 9a) at the 14th day. A slight disintegration of spheroids was also observed at the 7th and 14th day of treatment. Alternatively, free DOX and MTX reduced the diameter up to 30 µm (*p* < 0.005) without prominent spheroid disintegration. The cellular viability determined via the trypan blue assay at the 14th day indicated up to 57 ± 3.1% (*p* < 0.01) of cell death in treated spheroids compared to 37.5 ± 2.3 and 39.8 ± 1.5% in free DOX and MTX controls, respectively (Figure 9b).

In HT144 spheroids, CFO+DOX and ZFO+MTX, among DOX and MTX nanocarriers, produced a significant reduction in spheroid diameter (Figure 9a) at the14th day (423 µm, *p* < 0.005 and 168 µm, *p* < 0.0005, respectively) with 74 ± 8.9 and 81 ± 5.3% (*p* < 0.01) of cellular death (Figure 9b). The cells lost their compactness and started to disaggregate after three days of treatment, which increased with time (Figure 8). The average reduction in the diameter of spheroids after treatment with free DOX and MTX samples was 350 and 387 µm (*p* < 0.01) with 43 ± 2.3 and 39.7± 5.6% (*p* < 0.01) of cell death, respectively. The obtained results suggested better internalization of drug-loaded NPs compared to free drugs.

### Functionalized MFe_2_O_4_ NPs cause inhibition of MDR pump activity in treated HepG2 and HT144 cells

Due to the overexpression of P-glycoprotein (P-gp), cancer cells possess the ability to efflux chemotherapeutic drugs, a phenomenon known as MDR. P-glycoprotein belongs to the ABCB1 family of ABC proteins and is involved in the efflux of doxorubicin, paclitaxel, vincristine, rhodamine, and etoposide [63]. Conversely, multidrug resistance-associated protein 1 (MRP1) is a member of the ABCC1 family, responsible for the efflux of xenobiotics and hydrophobic drugs, namely methotrexate, vinca alkaloids, anthracyclines, antiandrogens, and heavy metals. Both multidrug resistance protein 1 (MDR1) and MRP1 proteins are majorly responsible for lowering the therapeutic outcomes of chemotherapy [64].

The present study was conducted to evaluate the role of drug-functionalized MFe_2_O_4_ NPs in hindering MDR pump activity in HepG2 and HT144 cells after a 24 h treatment at IC_50_ doses. The retention of the fluorometric dye was estimated relative to NTC (Figure 9c). Among DOX nanocarriers in HepG2, CFO+DOX and ZFO+DOX produced maximum significant inhibition of the MDR pump, with up to 6.8-fold (*p* < 0.005) dye retention compared to NTC. Similarly, among MTX nanocarriers, the highest dye retention of up to 4-fold was observed in CFO+MTX and ZFO+MTX (*p* < 0.05). Dye retention in free DOX and MTX was lower (3- and 1.9-fold, respectively). Cyclosporin A, used as positive control, caused inhibition of MDR up to 4-fold.

Similar results were obtained in HT144 cells. Among DOX nanocarriers, CFO+DOX and ZFO+DOX were responsible for the maximum inhibition of the MDR pump (up to 7-fold, *p* < 0.005), whereas dye retention in free DOX was 3.9-fold compared to NTC. Amidst MTX nanocarriers, ZFO+MTX and CFO+MTX proved to be the most efficient, with up to 6.5-fold (*p* < 0.01) dye retention compared to free MTX, with a lower dye retention of 4.1-fold.

The results indicated a possible role of drug-loaded CFO and ZFO NPs in combating MDR in cancer cells.

### Functionalized MFe_2_O_4_ NPs showed higher IC_50_ in normal cells as compared to cancer cells

The cytotoxicity of drug-loaded NPs was assessed in fresh human lymphocytes to determine their biocompatibility in vitro using the MTT assay. Freshly collected lymphocytes were exposed to varying concentrations (1, 10, 25, 50, and 100 µg/mL) of drug-loaded NPs for 24 h. Untreated cells and free drug controls (DOX and MTX) at 0.04, 0.4, 1, 2, and 4 µM (equivalent to drug attached with tested concentrations of NPs) were also included as controls.

**Figure 10:**
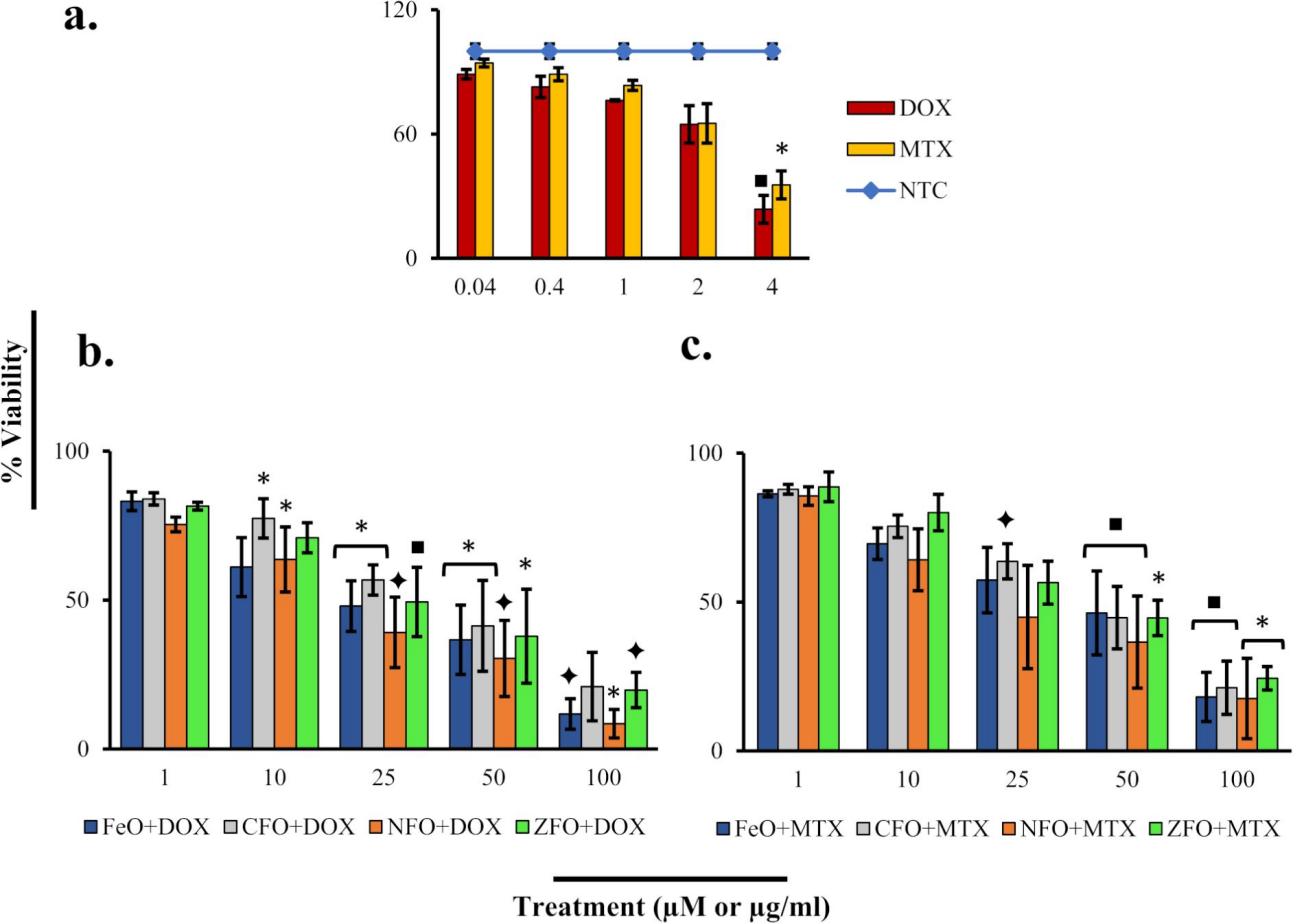
Dose-dependent cytotoxicity of fresh human lymphocytes treated with (a) free DOX and MTX (0.04, 0.4, 1, 2, and 4 µM equivalent to drug attached with NPs at tested doses) and NTC. (b) DOX-, and (c) MTX-loaded MFe_2_O_4_ (M = Fe, Co, Ni, Zn) NPs incubated for 24 h (1, 10, 25, 50, and 100 µg/mL). Plotted data indicates mean ± SD of three independent biological replicates with triplicates for all samples. *p* < 0.05 (one asterisk), *p* < 0.01 (black square symbol), *p* < 0.005 (black diamond symbol), paired two-tailed *t*-test when samples were compared to NTC.

The results indicated the cytotoxicity of drug-loaded NFO NPs in a dose-dependent manner, with percentage values of viability (highest to lowest) ranging from 75.37 ± 2.41 to 8.52 ± 4.75 in NFO+DOX and 85.56 ± 3.12 to 17.52 ± 9.46 in NFO+MTX. All NPs+DOX were significantly cytotoxic (*p* < 0.05) at 25 µg/mL and at higher concentrations (*p* < 0.05), excluding NFO which was cytotoxic at 10 µg/mL as well (*p* < 0.05). DOX-loaded CFO and ZFO were the least cytotoxic compared to other NPs with % viability values (from highest to lowest) ranging from 83.93 ± 2.01 to 20.91 ± 6.42 for CFO and 81.56 ± 1.33 to 19.74 ± 5.91 for ZFO at all doses (Figure 10b).

Among MTX-loaded NPs, all samples were significantly cytotoxic at 50 µg/mL and higher concentrations (*p* < 0.05). While NFO was cytotoxic (*p* < 0.005) even at 25 µg/mL, ZFO and CFO loaded with MTX were the most biocompatible with % viability values (from highest to lowest) ranging from 88.67 ± 5.01 to 24.33 ± 3.92 and 87.81 ± 1.60 to 21.26 ± 3.94, respectively (Figure 10c) at all tested doses. Free drug controls (DOX and MTX) showed significant cytotoxicity (*p* < 0.05) at the highest dose of 4 µM (Figure 10a).

The obtained results suggested an increased selectivity of nanocarriers towards cancer cells as previously reported [65], with IC_50_ values approximately 10–35 times higher (except NFO+DOX) in normal cells compared to cancer cells (Table 8).

## Conclusion

The present research describes sonochemically synthesized, biocompatible, highly colloidal, drug- (DOX and MTX) functionalized MFe_2_O_4_ (M = Fe, Co, Ni, Zn) NPs for in vitro anticancer drug delivery. All nanocarriers showed significantly increased (*p* < 0.005) drug release at an acidic pH value (pH 5.5) compared to that at a physiological pH value (pH 7.4), indicating their specificity towards cancer cells*.* In vitro cytotoxicity analysis indicated increased cytotoxicity in a dose-dependent manner compared to free drugs, with IC_50_ values ranging from 0.81–3.97 μg/mL in cancer cells and 18.35–43.04 μg/mL in normal cells. Similarly, cytotoxicity screening in 3D spheroids suggested a better internalization of drug-loaded NPs compared to free drugs. Most promising results were obtained in CFO and ZFO nanocarriers. Overall, NPs cause dose-dependent cytotoxicity via ROS generation, which causes genotoxicity, p53-mediated cell cycle arrest leading towards apoptosis. Furthermore, Ki-67 expression was highly inhibited (*p* < 0.005) in the presence of CFO+DOX and ZFO+DOX nanocarriers, indicating their anti-proliferative capability in cancer cells. In addition, CFO and ZFO nanocarriers showed significant (*p* < 0.05) inhibition of MDR pump activity in HepG2 and HT144 cells, suggesting their suitability for multidrug resistant cancers. Excellent colloidal stability, magnetic properties (coercivity = 883 and saturation magnetization = 56 emu/g), and specificity towards cancer cells support CFO nanocarriers as promising candidates for targeted cancer therapy domains. However, further investigations regarding pathway analysis, in vivo cytotoxicity, and magnetic-field-assisted drug delivery are needed.

## Experimental

### Materials

Iron nitrate [Fe(NO_3_)_3_·9H_2_O] and cobalt nitrate [Co(NO_3_)_2_·6H_2_O] (98%) were purchased from UNI-Chem. Zinc nitrate [Zn(NO_3_)_2_·6H_2_O)], nickel nitrate [Ni(NO_3_)_2_·6H_2_O], chloroform, and oleic acid (C_18_H_34_O_2_) (>99%) were purchased from Applichem. Poly(isobutylene-*alt*-maleic anhydride) (85%), dodecylamine (99%), tetrahydrofuran (THF) (99.9%), 1-ethyl-3-(3-dimethylaminopropyl)carbodiimide (98%), doxorubicin (99.9%), methotrexate (99.9%), Tris/borate/EDTA buffer (TBE, 98.8%), Dulbecco’s Modified Eagle’s Medium (DMEM, 99.9%), Roswell Park Memorial Institute (RPMI-1640, 99.9%) medium, GPPS (2 mM ʟ-glutamine, 1 mM Na pyruvate, 100 U/mL penicillin, 100 µg/mL streptomycin, 98%), Triton X-100 (lab grade), Trizma base, trypsin/EDTA (lab grade), sulforhodamine B (dye content 75%), ethidium bromide (lab grade), sodium dodecyl sulfate (SDS, lab grade), 2',7'-dichlorodihydrofluorescein diacetate (>97%) and 3-(4,5-dimethylthiazol-2-yl)-2,5-diphenyltetrazolium bromide (97.5%, lab grade), propidium iodide (>94%), acridine orange (>98%), RNAse A (>60%) and dimethyl sulfoxide 55% w/v (DMSO) were purchased from Sigma-Aldrich (USA). A DNA ladder (100 bp, research grade) and agarose (low-melting and normal) were purchased from Thermo Fischer Scientific and Hydra Gene Co., Ltd, respectively. Ethanol, trichloroacetic acid (TCA, 99%), trypan blue, fetal bovine serum (FBS, sterile filtered), orange G, sodium hydroxide (NaOH, 99%), and dibutylphthalate polystyrene xylene (DPX, research grade) mounting medium were purchased from Merck, Germany. Research-grade antigen retrieval solution (K8004), Ki-67 (clone MIB-1), p53 (clone DO-7) mouse monoclonal anti-human antibodies, horseradish peroxidase- (HRP) conjugated secondary antibody (DM822), peroxidase blocker (DM821, lab grade), diaminobenzidine (DAB), chromogen (DM827), and hematoxylin (K8018) ready to use were obtained from Agilent Technologies, Inc. (USA).

### Colloidal synthesis of MFe_2_O_4_ (M = Fe, Co, Ni, Zn) nanoparticles

The two-step sonochemical method was used for the synthesis of MFe_2_O_4_ (M = Fe, Co, Zn, Ni) NPs. The coprecipitation method was used in the first step of the synthesis of MFe_2_O_4_ nanoparticles (0.2 M) by mixing iron nitrate [Fe(NO_3_)_3_·9H_2_O] and (Co/Zn/Ni) nitrates with a molar ratio of Fe/M (2:1) in 100 mL of deionized water. The solution was stirred for 15 min, heated at 70 °C, and further stirred for 1 h after the addition of NaOH (3M) which settled down forming precipitates. The precipitates were washed four times and collected with the help of a magnet. The samples were dried in the oven, annealed at 600 °C, and redispersed in oleic acid (1:3) by sonication for 4 h. The resultant precipitates were washed with methanol and resuspended in chloroform [30].

### Physical characterizations

Structural studies were carried out at an XRD D8-Advance Bruker AXS diffractometer with Cu Kα radiation (λ = 1.54 Å). The Debye–Scherrer formula (Equation 1) was used to calculate the average crystallite size of NPs from the XRD peak of the (311) plane [30]:


[1] $$D=\frac{K\lambda }{\beta \cos\theta },$$

where *D* is the average crystallite size, *K* = 0.94, λ = 1.54 Å is the X-ray wavelength, β represents the full width at half maximum (FWHM), and θ represents the Bragg's diffraction angle.

The surface morphology and major elemental composition were obtained by high-resolution transmission electron microscopy (JEM 2100F) and energy dispersive spectroscopy (TESCAN-VEGA3), respectively. The magnetic behavior was determined by using a physical property measurement system (Quantum Design, USA). The colloidal stability and hydrodynamic size of NPs were studied by using a Zetasizer Nano ZS (Malvern Instruments, 69 UK) and the uniform size distribution by gel electrophoresis (GE BIORAD). Drug attachment and drug release analyses were performed by using UV–vis spectroscopy (Thermo Scientific Evo 220).

### Phase transfer, polymer coating, and purification of MFe_2_O_4_ (M = Fe, Co, Ni, Zn) NPs by gel electrophoresis

The synthesis and polymer coating of NPs were carried out as previously described [22,30]. The nanoparticles (1 mL) were mixed with 350 µL of PMA (0.8 M) and stirred at 60 °C for 1 h. The samples were slowly dried under vacuum and finally redispersed in SBB pH 9. PMA-coated samples were filtered using a 0.2 µm syringe filter and concentrated using centrifugal filters (Amicon Ultra-4). The concentrated samples were purified by using 1% agarose gel at 100 V for 90 min. Discrete NP bands on the gel were cut and extracted using a 50 kDa dialysis membrane (Spectrum Laboratories, Inc.) in TBE buffer [66]. Finally, the gel-extracted NPs were concentrated by using centrifugal filters and resuspended in SBB (pH 9.0).

### Preparation of drug-loaded nanoparticles

The purified PMA-coated MFe_2_O_4_ (M = Fe, Co, Zn, Ni) NPs were further modified with DOX and MTX via EDC chemistry. The NPs were incubated with optimized concentrations of EDC and drug (DOX and MTX) for 2 h at room temperature. The drug attachment to the surface of NP was confirmed by UV–vis spectroscopy [30]. The drugs unbound from the samples were removed by 50 KDa centrifugal filters and their concentration in the waste was confirmed with the help of drug titration curves. Drug encapsulation efficiency (EE) and drug-loading capacity (LC) were determined using the following equations [67]:


[2] $$\text{EE}\%=\left(\frac{\begin{array}{l} \text{absorbance of drug used}-\\ \text{absorbance of waste} \end{array}}{\text{absorbance of drug used}}\right)\times 100.$$

[3] $$\text{LC}\%=\left(\frac{\text{entrapped drug}}{\text{nanoparticle weight}}\right)\times 100.$$

**Figure 11:**
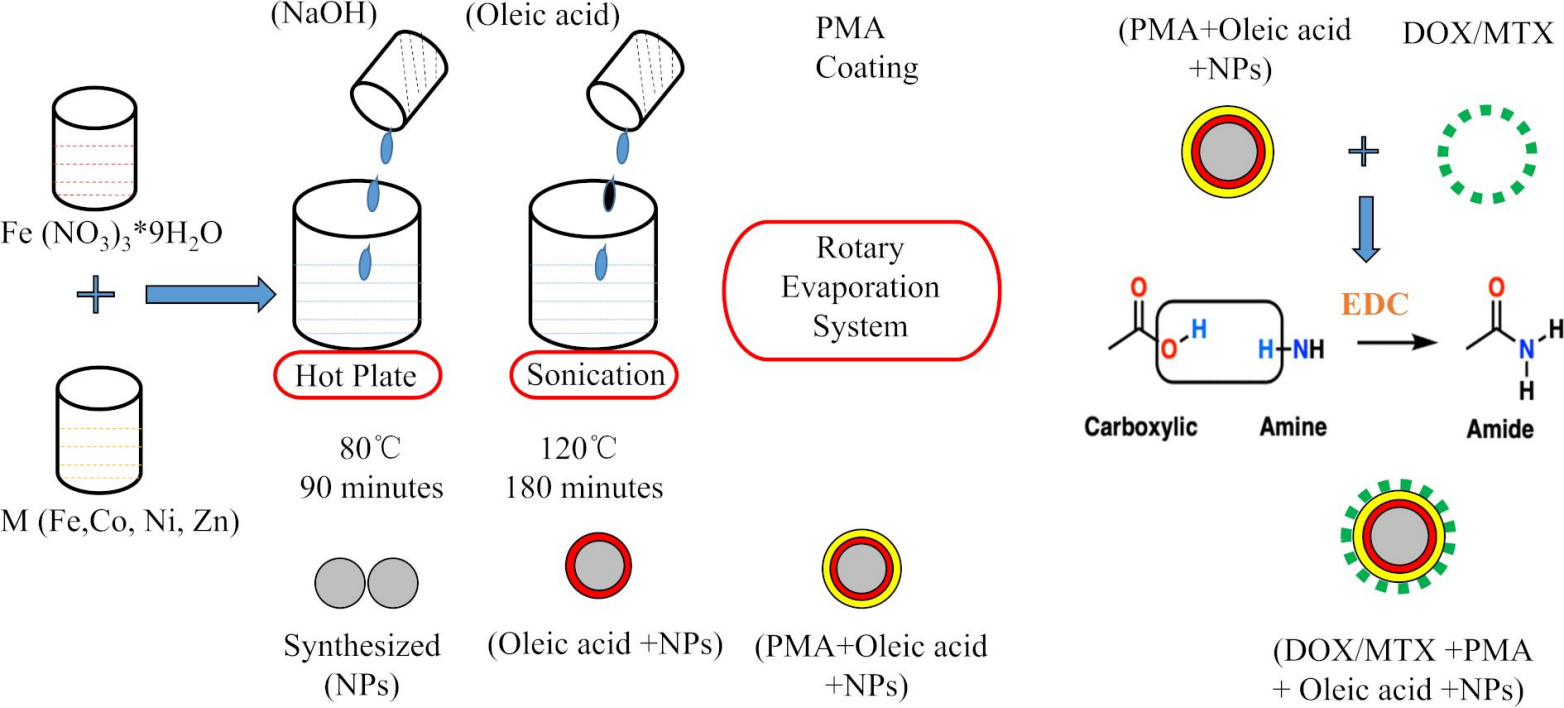
Graphical representation of sonochemical synthesis, PMA-coating, and drug (DOX and MTX) functionalization of MFe_2_O_4_ (M = Fe, Co, Zn, Ni) NPs.

Figure 11 indicates a graphical representation of the complete synthetic route of drug-functionalized NPs.

### pH-dependent drug release kinetics

Drug release kinetics of DOX- and MTX-loaded MFe_2_O_4_ (M = Fe, Co, Zn, Ni) NPs were studied at different pH values [68]. The nanoparticles were dispersed in PBS with pH values ranging from 5.5–7.4 and spectrophotometric data were measured at different time intervals (0, 1, 5, 10, 20, 40, 60, and 120 min). After every time point, the samples were centrifuged at 10000 rpm for 5 min and the supernatants were analyzed by spectrophotometry. UV–vis readings were analyzed with the help of DOX and MTX titration curves to estimate the amount of released drugs. The percentage of drug release was calculated by the formula given in Equation 4:


[4] $$\text{Drug release }\%=\left(\frac{\text{absorbance of supernatant}}{\text{absorbance of drug-loaded NPs}}\right)\times 100.$$

### Cell culture

The cell lines human malignant melanoma (HT144, ATCC^®^ HTB-63™) and human hepatocellular carcinoma (HepG2, ATCC^®^HB-8065™) were used in this study. The cells were grown in DMEM supplemented with 10% FCS and 1% GPPS in a humidified incubator (37 °C) with 10% CO_2_. The cells were harvested using trypsin/EDTA (0.5 mM) for 1 min at room temperature.

### In vitro cytotoxicity screening of drug-loaded MFe_2_O_4_ (M = Fe, Co, Ni, Zn) NPs

The cytotoxicity screening of colloidal drug-loaded MFe_2_O_4_ (M = Fe, Co, Ni, Zn) NPs was performed by using the SRB assay in vitro [30]. HepG2 and HT144 cells (>90% viability, 1.5 × 10^5^ cells/mL) were seeded onto 96-well plates (Falcon*^®^* 96*-*well, flat-bottom*,* clear microplate) and treated with 5 µg/mL of drug-loaded NPs for 24 h at 37 °C, followed by fixation with 50% trichloroacetic acid (TCA) for 60 min at 4 °C. The plates were washed thrice with deionized water to remove TCA and then air dried. Afterwards, the SRB dye (0.05%) was added at room temperature for 30 min to stain the cells. The excess dye was washed with 1% acetic acid 4–5 times. After the plates were air dried, photographs were taken at a 200× magnification with an Olympus CK2 light microscope with an attached camera (Optika C-B10 digital camera) and analyzed using the Optika Pro View software (version x86, 3.7.13977.20190224). The experiment was performed twice with triplicates for all samples. The experimental controls included untreated cells, free doxorubicin, free methotrexate, and NPs-PMA.

### Determining the IC_50_ concentration of drug-loaded MFe_2_O_4_ (M = Fe, Co, Ni, Zn) NPs

In order to determine the metabolic activity and half maximal inhibitory concentration (IC_50_) of drug-loaded NPs, the MTT assay was used [66]. HepG2 and HT144 cells (>90% viability, 1.5 × 10^5^ cells/mL) were exposed to varying concentrations (1, 10, 25, 50, and 100 µg/mL) of NPs for 24 h followed by the addition of MTT (0.5 mg/mL) and further incubation for 4 h. The MTT reagent was solubilized overnight by using 10% acidified SDS. Control groups included NPs-PMA, NTC, DOX (10 μM), and MTX (10 μM). Non-cellular controls included replicates of media only and NPs only. The absorbance at 565 nm was measured using a microplate reader (FLUOstar Omega microplate reader, BMG LABTECH). The percentage values of cellular viability were calculated by using the following formula:


[5] $$\%\text{ viability}=\left(\frac{\begin{array}{l} \text{absorbance of sample}-\\ \text{absorbance of sample control} \end{array}}{\begin{array}{l} \text{absorbance of NTC}-\\ \text{absorbance of media only} \end{array}}\right)\times 100.$$

The IC_50_ values were calculated by using the following formula:


[6] $${\text{IC}}_{50}=\frac{b-\left[\left(b-a\right)\left(50\%-d\right)\right]}{\left(c-d\right)},$$

where *a* and *b* are the drug concentration values which yield just more than 50% and just less than 50% of viable cells, respectively, *c* is the percentage of viability yielded by the drug concentration value *a*, and *d* is the percentage of viability yielded by the drug concentration value *b*. The experiment was repeated twice with triplicates for each sample.

### Determination of necrotic/apoptotic cells via fluorescence microscopy

In order to determine the extent of apoptosis and necrosis in treated cells, AOPI staining was used as previously described [30]. Pre-seeded HepG2 and HT144 cells (>90% viability, 1.0 × 10^5^ cells/mL) were treated with drug-loaded NPs (5 and 10 µg/mL) for 3 h under appropriate culture conditions. Control groups included NTC, NPs-PMA (10 μg/mL), free DOX and free MTX (5 μM each). Afterwards, the cells were washed with 1× PBS and stained with AOPI (100 and 32 μg/mL) for 1 min at room temperature and visualized under a fluorescence microscope (200×, Nikon, MicroPhot-SA). Green fluorescence indicates viable cells, red fluorescence indicates necrotic cells, whereas yellow to orange fluorescence indicates early and late apoptotic cells, respectively. By using an Optika Pro View (version x86, 3.7.13977.20190224) instrument, live, necrotic, and apoptotic cells were counted, and their percentages were calculated relative to NTC.

### Determination of oxidative stress in treated cells

The cell-permeant H_2_-DCFDA was used to determine the extent of ROS production in HepG2 and HT144 cells upon treatment with drug-loaded NPs over a period of time (0–45 min) [69]. Briefly, cells were seeded onto a 96-well plate at a density of 1.5 × 10^5^ cells/mL under appropriate culture conditions. After 24 h, media were removed, replaced with PBS containing 2% FCS and 25 μM H_2_-DCFDA, and incubated for 45 min. Cells were treated with NPs at IC_50_ concentrations and the fluorescence intensity values were recorded using a microplate reader (FLUOstar Omega microplate reader, BMG LABTECH) at various time points and at emission and excitation wavelengths of 355 and 590 nm, respectively (gain = 700). The control groups included free DOX, free MTX (0.2 μM each), and NTC. Non-cellular controls included NPs only and DCF only samples. The experiment was performed in triplicates.

### Cell cycle analysis

The cell cycle analysis was performed by flow cytometry. Pre-seeded HepG2 and HT144 cells (>90% viability, 1.5 × 10^5^ cells/mL) were treated with IC_50_ doses of drug-loaded NPs for 24 h. Control groups included NTC and free drugs (DOX and MTX, 0.2 μM each). The cells were harvested, washed with PBS, and fixed in fixative solution containing 70% ethanol, 10% PBS, and 20% deionized water at 4 °C. For flow cytometry, fixed cells were washed with PBS and incubated with a staining solution containing 50 μg/mL of PI and 100 µg/mL of RNase A for 30 min at room temperature in the dark. The analysis of at least 10000 cells was performed using a flow cytometer (CytoFLEX LX, Beckman Coulter Life Sciences) and the CytExpert software (Version 2.4) [70].

### Detection of DNA strand breaks in treated cells: the alkaline comet assay

A single-cell gel electrophoresis (alkaline comet assay) was performed as described earlier [70]. HepG2 and HT144 cells (>90% viability) were exposed to drug-loaded NPs at IC_50_ doses for 1 h under standard culture conditions. Control groups included NTC and free drugs (DOX and MTX, 0.2 μM each). The cells were harvested, counted, embedded in 0.7% low-melting agarose, and spotted on comet assay slides. After solidification on ice, the slides were immersed into cell lysis buffer (2.5 M NaCl, 100 mM Na_2_-EDTA, 10 mM Trizma base pH 10, 1% sodium sarcosinate, and 1% Triton-X100) overnight at 4 °C. In the following day, the slides were immersed into a pre-chilled alkaline solution (0.3 M NaOH and 1 M Na_2_-EDTA, pH 13) for 20 min to allow for DNA unwinding and electrophoresed for 20 min at 25 V and 300 mA. After air drying, staining was performed using PI (5 µg/mL in PBS) and at least 150 cells were analyzed for each sample using the ImageJ software to calculate median olive tail moment values relative to NTC.

### Detection of DNA fragmentation in treated cells

DNA fragmentation is a hallmark of cellular apoptosis resulting in the formation of small DNA fragments of 180 bp (or multiple) which can be visualized on agarose gel [71]. A ladder assay was performed using the DMSO method as described previously [72]. Briefly, HepG2 and HT144 cells (>90% viability, 1.0 × 10^5^ cells/mL) were treated with IC_50_ doses of drug-loaded NPs for 24 h at standard culture conditions. The media were removed, cells were washed with PBS and collected via trypsinization. Cellular lysis was performed by adding DMSO (100 µL) to the pellets, which were mixed by vortexing. An equal volume of TE buffer (pH 7.4) containing 2% SDS was added and the samples were vortexed. The samples were then centrifuged at 12000 rpm for 10 min and the resulting supernatant fractions containing low molecular weight DNA fragments were quantified using the Nanodrop 2000C. Equal amounts of DNA from all samples were electrophoresed on a 2% agarose gel (containing ethidium bromide at 50 µg/mL) along with orange G dye for 2 h at 50 V. The gel was visualized by using a UV transilluminator and the results were recorded. The control groups included NTC, DOX, and MTX (30 µM).

### Expression assessment of Ki-67 and p53 cancer biomarkers via immunocytochemistry

Ki-67 and p53 protein expression was evaluated by ICC [73] (Dako EnVision^TM^ FLEX detection system). HepG2 and HT144 (>90% viability, 1.5 × 10^5^ cells/mL) cells were cultured on sterile coverslips in 24-well plates. Cells were treated with IC_50_ doses of drug-loaded NPs for 24 h, followed by fixation with TCA and washing with deionized water. The cells were immersed in an antigen retrieval solution at 95 °C for 45 min. Endogenous peroxidases were blocked by adding a peroxidase blocker for 10 min. Ki-67 (clone MIB-1, working dilution 1:150) and p53 (clone DO-7, working dilution 1:50) mouse monoclonal antibodies were then added, and the cells were incubated at 4 °C overnight followed by the addition of HRP-conjugated secondary antibody (rabbit, polyclonal) for 30 min and DAB chromogen for 10 min to obtain the desired dark brown stain with washings in between. The cells were counter stained with hematoxylin, dehydrated, mounted, and observed under a light microscope (Nikon, MicroPhot-SA) at a 200× magnification with an attached camera (Optika C-B10 digital camera) and analyzed by using the Optika Pro View software (version x86, 3.7.13977.20190224). The percentage of antibody positive cells was calculated using the following formula:


[7] $$\%\text{ antibody positive cells}=\left(\frac{\begin{array}{l} \text{number of }\\ \text{antibody positive cells} \end{array}}{\text{total number of cells}}\right)\times 100.$$

### Cytotoxicity assessment of drug-loaded MFe_2_O_4_ (M = Fe, Co, Ni, Zn) NPs in HepG2 and HT144 3D spheroids

Cancer cells grown in 3D cultures called spheroids, closely resemble their in vivo phenotype. HepG2 and HT144 spheroids were treated with drug-loaded NPs (5 µg/mL) for 14 days to assess their cytotoxicity in 3D culture models. Control groups included NTC and free drugs (DOX and MTX, 0.2 µM each). In brief, HepG2 and HT144 cells (>90% viability, 5000 cells/well) were seeded onto sterile, agarose-coated (1.5% prepared in autoclaved deionized water; 50 µL/well) 96-well plates (Falcon*^®^* 96*-*well, flat-bottom*,* clear microplate) with 200 µL medium/well. The plates were centrifuged at 2500 rpm for 5 min to allow cellular accumulation in the agarose meniscus. The plates were incubated at 37 °C for three days to allow the formation of closely packed 3D spheroids prior to treatment. The media were changed after every 48 h [74]. At every time point, photographs were captured at a 100× magnification using an Olympus CK2 light microscope with an attached camera (Optika C-B10 digital camera) and analyzed using the Optika Pro View software (version x86, 3.7.13977.20190224). The average diameter of the spheroids was determined using the ImageJ software.

At the 14th day of treatment, the spheroids were collected, washed with PBS, and trypsinized for 5 min to obtain a suspension with single cells. The cellular viability was then determined in triplicates using the trypan blue method [75].

### Assessment of multidrug resistance pump activity in treated HepG2 and HT144 cells

HepG2 and HT144 exhibit intrinsic expression of ATP-binding cassette (ABC) transporters responsible for inducing multidrug resistance in response to chemotherapy [76–77].

Here, a fluorometric MDR assay kit (ab 112142, Abcam, Cambridge, MA, USA) was used to determine MDR1 and MRP1 activity in HepG2 and HT144 cells using the protocol suggested by the manufacturer [78]. In brief, cells (>90% viability, 1.5 × 10^5^ cells/mL) were treated with drug-loaded NPs at IC_50_ doses for 24 h. Free drug controls (DOX and MTX, 0.2 µM each) and NTC were included as controls. Cyclosporin A (10 µM) was included as a positive control. After treatment, the plates were incubated at room temperature with a dye loading solution (100 µL/well) for 3 h in the dark. Fluorescence intensity relative to NTC was determined after subtracting the drug only background at 485/530 nm using a plate reader (FLUOstar Omega microplate reader, BMG LABTECH). The higher the cellular fluorescence, the higher the MDR pump inhibition. The experiment was performed in triplicates for all samples.

### IC_50_ of drug-loaded MFe_2_O_4_ (M = Fe, Co, Ni, Zn) NPs in normal cells: biocompatibility assessment

The cytotoxicity of drug-loaded NPs was evaluated in fresh lymphocytes in vitro. Fresh peripheral blood (5 mL) was collected from healthy individuals in EDTA vacutainers under informed consent. The blood was diluted (1:3) with RBCs lysis buffer (155 mM NH_4_Cl, 0.1 mM EDTA, and 10 mM KHCO_3_; pH 7.2) and incubated at room temperature for 5 min with mixing in between, followed by centrifugation at 2000*g* for 5 min. The process was repeated 5 times to obtain a clear pellet of lymphocytes [79]. The lymphocytes were resuspended in RPMI-1640 medium containing 10% FCS and 1% GPPS. Cell viability was assessed via the trypan blue method [75].

IC_50_ concentrations of drug-loaded NPs were calculated by performing an MTT assay as described previously.
